# Socioeconomic position and the gut microbiota: a narrative synthesis of the association and recommendations

**DOI:** 10.1080/19490976.2026.2623356

**Published:** 2026-01-31

**Authors:** Jasmine Samantha Ratcliff, Meena Kumari, Patrick Varga-Weisz, Rick O’Gorman

**Affiliations:** a Institute for Social and Economic Research, University of Essex, Colchester, UK; b School of Life Sciences, University of Essex, Colchester, UK; c International Laboratory for Microbiome Host Epigenetics, Department of Genetics, Evolution, Microbiology, and Immunology, Institute of Biology, University of Campinas, Campinas, Brazil; d Department of Psychology, University of Essex, Colchester, UK

**Keywords:** Microbiome, microbiota, microbiology, gut microbiome, social determinants of health, socioeconomic position, socioeconomic factors, public health

## Abstract

Evidence suggests that socioeconomic position (SEP) may shape the gut microbiota (GM), representing a mechanism through which social and environmental factors may drive health inequalities, yet no systematic review has examined this association. In this narrative systematic review, we searched PubMed, Web of Science, and Scopus up to 30 November 2024 for observational studies examining associations between measures of SEP and GM diversity, composition, or function in participants of any age, ethnicity, or location. We identified 1,479 unique studies, of which 26 met the inclusion criteria for this review. Associations were observed between SEP indicators and GM features, including alpha (*α*) and beta (*β*) diversity, taxonomic composition, and functional pathways. Notably, socioeconomic patterns in *α*-diversity differed by context, with greater diversity observed in advantaged groups in high-income countries (HICs) but in disadvantaged groups in low- and middle-income countries (LMICs). Differences in *β*-diversity suggest that advantaged and disadvantaged groups have distinct GM profiles. Furthermore, considerable heterogeneity was evident across studies, particularly in sampling, sequencing, and analytical methods. Overall, socioeconomic-related differences in the GM are evident globally, highlighting the microbiota as a potential target for interventions aimed at reducing health disparities. Further research employing larger and more diverse cohorts, longitudinal designs, metagenomic sequencing approaches, and comprehensive measurement and adjustment of key covariates is needed to deepen understanding of this relationship.

## Background

The gut microbiome (GM)—a complex ecosystem of trillions of microorganisms, including bacteria, viruses, fungi, and archaea—influences many aspects of human physiology via neural, hormonal and immunological pathways. The composition of the GM changes over the lifespan, with recent research emphasising that environmental factors often exert a stronger influence on microbial composition than genetics.^1^^,^^2^ Given that the GM is greatly influenced by environmental factors, it has the potential to serve as a mechanism through which social, political, and economic conditions may contribute to health disparities.^3^^,^^4^

Socioeconomic position (SEP), a person’s rank in the social hierarchy shaped by factors like education, occupation, and income, is one of the most significant social determinants of health (SDoH)^5^. There is robust evidence for a socioeconomic gradient in health, whereby individuals from disadvantaged SEP experience poorer health outcomes. SEP shapes a range of interconnected factors that can get “under the skin” to influence human biology, with emerging research identifying the GM as a potential mediator in the relationship between SEP and health.^6^ SEP has the potential to influence the GM through multiple interconnected pathways, including psychosocial stressors, social networks, health behaviours, early-life exposures, and characteristics of the built environment.^7^^,^^8^ The human microbiome can be viewed as a biological reflection of the SDoH, encapsulating both current and early-life socio-environmental conditions, as evidenced by shifts in GM composition following migration from non-industrialised to industrialised environments.^9^^,^^10^

The GM changes across the life course due to developmental, dietary, and environmental factors. During the “first 1000 days” after birth, rapid colonisation occurs—a critical period for immune and metabolic development shaped by delivery mode, breastfeeding, and solid food introduction, with disruptions potentially causing long-term health consequences.^11^ The infant microbiota stabilises into an adult-like composition around age three. Whether a distinct paediatric microbiome exists beyond infancy remains unclear, with conflicting evidence on taxonomic differences between children and adults.^12^^,^^13^ The adult microbiome is relatively stable with an established functional core, though susceptible to disruption by dietary changes, infections, and antibiotics.^11^ In older age, reduced physical activity and immune dysregulation associate with dysbiosis, including pathogen overgrowth and reduced *α*-diversity.^14^ SEP measurement also varies by life stage: infant and childhood studies capture household or parental SEP, while adult studies assess individual-level measures (e.g., education, occupation, and income), representing fundamentally different socioeconomic dimensions that do not fully align with biological developmental stages.

To our knowledge, no systematic review has yet synthesised the evidence on the association between SEP and the GM, a research area that has gained momentum in the past five years due to advances in sequencing technologies and bioinformatic approaches. This systematic review provides a comprehensive overview of the growing global literature on the relationship between individual- and neighbourhood-level SEP and GM features, including diversity, composition, and function. We included studies from both high-income (HICs) and low- and middle-income countries (LMICs), as SEP is experienced differently across these contexts, and research shows notable differences in GM between them.^15^ Given that the GM differs across age and geography, assessing diverse populations is essential to understanding the functional and health implications of socioeconomic-driven changes in microbiome composition.

## Methods

This systematic review adheres to the relevant criteria of the PRISMA statement^16^ and was registered on PROSPERO (CRD42024622112).

### Search strategy and selection criteria

We systematically searched PubMed, Web of Science, and Scopus for articles from database inception to 30 November 2024. Only peer-reviewed, full-text studies in English were eligible. Three main search terms were used: “microbiota”, “socioeconomic”, and “sequencing”, including related MeSH terms and keywords. Additional studies were identified via references of relevant articles.

Inclusion criteria for articles were: (1) observational studies (cross-sectional, case-control, cohort); (2) participants of any age, location, or ethnicity; (3) amplicon (e.g., 16S rRNA) or metagenomic sequencing of faecal samples; (4) at least one socioeconomic factor as exposure (e.g., income, wealth, occupation, education, neighbourhood-level SEP); and (5) GM composition and/or function as outcome (taxonomic composition, α/β-diversity, functional pathways, or enterotypes). A visual overview of the GM outcomes examined is provided in Figure S1 (supplementary materials).

### Screening and data extraction

Titles and abstracts were screened using Rayyan.^17^ The primary reviewer (JR) screened all studies; secondary reviewers (PVW, MK) each independently screened a randomly selected 10% subset. Studies meeting criteria or with unclear eligibility proceeded to full-text screening, conducted by JR (100%) and PVW/MK (10% each, randomly selected). Conflicts were resolved by discussion or a third reviewer. Reasons for exclusion at the full-text stage were documented. JR extracted data from included studies. Extracted data from articles included: title; authors; country; country income classification (HIC/LMIC); publication year; study design; sample size; sample characteristics; sample collection/processing details (e.g., storage, preservation, DNA extraction); socioeconomic variables; microbiota assessment methods; bioinformatics pipelines; GM characterisation methods (α/β-diversity, taxa, pathways); associations between SEP and microbiome (including direction of association); statistical methods; confounders measured; and raw data availability.

### Assessment of methodological quality

Study quality was assessed using the National Heart, Lung, and Blood Institute’s Study Quality Assessment Tools.^18^ JR assessed all studies; PVW and MK each independently assessed 25% of studies (randomly selected) to verify consistency in quality ratings. As the tools lack predefined cutoffs, studies were holistically rated as “Good” (minimal bias, robust methods), “Fair” (some limitations not compromising core findings), or “Poor” (substantial flaws limiting validity). Age, sex, and BMI were considered key confounders given their associations with both SEP and microbiome composition. The complete quality assessment is provided in the supplementary materials (Table S4 and S5).

## Results

**Figure 1.**
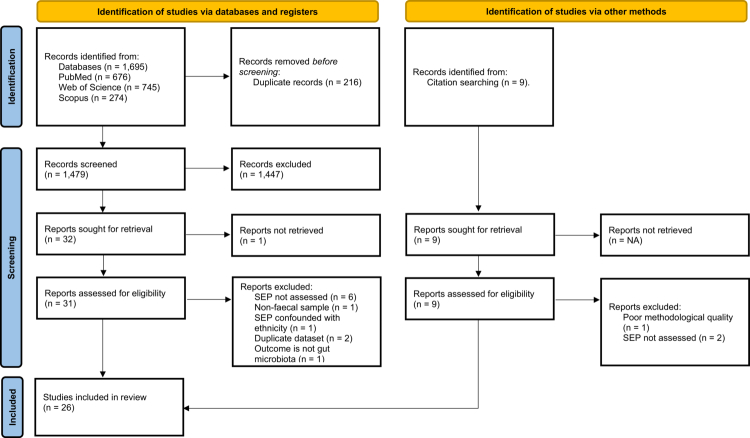
PRISMA flowchart depicting the process used to determine study inclusion. We systematically searched PubMed, Web of Science, and Scopus for articles from database inception to 30 November 2024. Only peer-reviewed, full-text studies in English were eligible. Three main search terms were used: “microbiota”, “socioeconomic”, and “sequencing”, including related MeSH terms and keywords. Additional studies were identified via references of relevant articles. A total of 26 studies met the inclusion criteria and were included in the review.

Our search yielded a total of 1479 potentially relevant studies (after 216 duplicates were removed). After initial screening, 40 studies were selected for full-text review (Figure 1), of which 26 studies met criteria for inclusion in the systematic review (Table 1).

**Table 1.** Summary of studies included in the systematic review.

Study	Design	Location	Sample size	HIC/LMIC	Life stage	Age (mean ± SD) (where available)	% Female	SEP exposure	Sequencing method	Quality rating	Raw data availability
Amaruddin et al.^19^	Cross sectional	Makassar, Indonesia	140	LMIC	Childhood	10.33 ± 0.85	60.7	Disadvantaged SEP school vs advantaged SEP school	16S rRNA	Poor	N/A
Balakrishnan et al.^20^	Cross sectional	Alabama, USA	60	HIC	Childhood	8.56 ± 1.41	58.3	Household income Parental education	16S rRNA	Fair	N/A
Bishehsari et al.^21^	Cross sectional	Chicago, USA	123	HIC	Adulthood	43.4	65.4	Neighbourhood SEP composite	16S rRNA	Fair	SRA: SRP133159 BioProject: PRJNA434249
Bowyer et al.^6^	Cross sectional	Cohort: TwinsUK, UK	799-1672 depending on SES variable	HIC	Adulthood	61.9 ± 11.2	91.0	Household Income Education Index of Multiple Deprivation (IMD)	16S rRNA	Good	N/A
Chong et al.^22^	Cross sectional	Cohort: DIAMOND, Auckland, New Zealand	213	HIC	Infancy	Collection at 10 days D10 and 4 months 4 M	16S rRNA: D10: 43.7 4 M: 42.1 Metagenomics: 4 M: 38.3	Census-based New Zealand geography deprivation index (NZDep2013) using parents' self-reported postcode	16S rRNA	Fair	16S rRNA: BioProject: PRJNA645223 Metagenomics: BioProject: PRJNA648487
Flannery et al.^23^	Cross sectional	USA	40	HIC	Childhood	6.1 ± 0.69	57.5	Life Events Checklist	Shotgun metagenomics	Fair	BioProject: PRJNA496479
Gacesa et al.^1^	Cross sectional	Cohort: Dutch Microbiome Project (DMP), Northern Netherlands	8208	HIC	Adulthood (6% children aged 8-17)	48.4 ± 14.8	57.4	Monthly individual income Neighbourhood income	Shotgun metagenomics	Good	European Genome-Phenome Archive: EGAS00001005027
Galley et al.^24^	Cross sectional	Columbus, Ohio, USA	77 Mother-child pairs	HIC	Infancy	Mothers at delivery: 31.1 ± 5.43 Children: 23.1 months ± 2	46.8	Household income Parental education	16S rRNA	Fair	SRA: SRP045568
Gschwendtner et al.^25^	Cross sectional	Cohort: LISA birth cohort, Germany	166	HIC	Childhood	6	44	Maternal education	16S rRNA	Fair	N/A
Gul et al.^26^	Cross sectional	Pakistan	117	LMIC	Adulthood	28.7 ± 5.45	52.1	Socioeconomic class dictated by individual income data	16S rRNA	Poor	ENA: PRJEB59240
He et al.^27^	Cross sectional	Cohort: Guangdong Gut Microbiome Project, China	6896	LMIC	Adulthood	52.8 ± 14.7	55.2	Yearly individual income Yearly individual spending	16S rRNA	Fair	ENA: PRJEB18535
Kaplan et al.^9^	Cross sectional	USA - Chicago, Miami, Bronx, San Diego	1674	HIC	Adulthood	57.1	64.2	Education Parental high school education Household income	16S rRNA	Fair	ENA: ERP117287
Kemp et al.^28^	Cross sectional	Alabama, USA	136	HIC	Childhood	12.1	48	Composite SES variable (average of standardised household income and parental education)	16S rRNA	Fair	N/A
Klee et al.^29^	Cross sectional	Cohort: Luxembourg Parkinson's Study (LUXPARK), Luxembourg	258 (58 with mild cognitive impairment)	HIC	Adulthood	64.6 ± 8.3	39.5	Education	16S rRNA	Fair	N/A
Kortekangas et al.^30^	Cross sectional	Malawi, Africa	579	LMIC	Infancy	Samples collected at 1, 6, 12, 18, 30 months (children) and 1 month after delivery (mothers)	N/R	Household assets index	16S rRNA	Fair	ENA: PRJEB29433
Kwak et al.^31^	Cross sectional	Cohort: Food and Microbiome Longitudinal Investigation (FAMiLI), New York, USA	825	HIC	Adulthood	59.6 ± 11.1	63.3	Social Deprivation Index (SDI) Occupational Socioeconomic Index Education Neighbourhood income	16S rRNA	Fair	SRA: PRJNA559143
Lapidot et al.^32^	Cross sectional	Israel	139	HIC	Childhood	11.42	41	SEP composite score: residential SES rank, number of parental schooling years, household crowding index	16S rRNA	Fair	N/A
Lapidot et al.^33^	Cross sectional	Israel	176	HIC	Childhood	7.7 ± 0.9	40.3	Village SEP rank Household crowding index	16S rRNA	Fair	N/A
Levin et al.^34^	Cross sectional	Cohort: WHEALS birth cohort, Michigan, USA	298	HIC	Infancy	Neonates: median: 1.2 (min 0.5, max 4.6) Infants: Median: 6.6 (min 5.6, max 10.6)	46.8	Household income Education	16S rRNA	Fair	ENA: PRJEB13896
Lewis et al.^35^	Cross sectional	USA	588	HIC	Childhood	4.5 ± 3.63	45	Parental education	Shotgun metagenomics	Poor	NCBI Genbank: PRJNA695570
Miller et al.^36^	Cross sectional	Chicago, USA	41 sigmoid biopsies, 26 faecal samples	HIC	Adulthood	39.1 ± 14.2	61.4	Neighbourhood SEP composite	16S rRNA	Fair	SRA: PRJNA287290
Moran-Ramos et al.^37^	Cross sectional	Mexico	926	LMIC	Childhood	9.93	49.3	Parental education Monthly household income Household size	16S rRNA	Fair	N/A
Mosites et al.^38^	Cross sectional	Kenya	184	LMIC	Adulthood	39.7 months ± 13.3	60	Asset-based index	16S rRNA	Poor	SRA: SRP090785
Mulder et al.^39^	Cross sectional	Cohort: Generation R Study, Rotterdam, Netherlands	2004	HIC	Childhood	9.8 ± 0.3	49.6	Maternal education Financial difficulties Neighbourhood problems	16S rRNA	Good	N/A
Shridhar et al.^8^	Cross sectional	Western Honduras	1871	LMIC	Adulthood	41 ± 17	63.7	Spending per month and travel spending Education Household wealth score	Shotgun metagenomics	Good	SRA: PRJNA999635
Zuniga-Chaves et al.^40^	Cross sectional	Cohort: Survey of the Health of Wisconsin (SHOW), Wisconsin, USA	721	HIC	Adulthood	54.9 ± 16.1	57.6	Economic Hardship Index (EHI)	16S rRNA	Good	SRA: PRJNA999362

All included studies were cross-sectional; one study collected infant GM samples at two time points but examined SEP–microbiome associations cross-sectionally rather than longitudinally.^22^ 19 studies were in HICs and seven in LMICs. Geographically, 11 studies were from the USA, five in Europe, five in Asia, two in Africa, and three across North America and New Zealand (Table 1). Regarding age distribution, 12 studies examined adults (18+; 8 HICs, 4 LMICs), 10 examined children aged 3−13 (8 HICs, 2 LMICs), and 4 examined infants aged 0−2 (3 HICs, 1 LMIC). One study included participants aged 8-84 but was classified as an adult study because children comprised only 6% of the sample^1^. Two studies were from the same research group and based on the same population, with some possible participant overlap.^32^^,^^33^ Sample sizes ranged from 41 to 8,202 participants.

22 studies used 16S rRNA amplicon sequencing for GM analysis; one also used shotgun metagenomics.^22^ Four studies employed shotgun metagenomic sequencing.^1^^,^^8^^,^^23^^,^^35^ All studies reported at least partial specimen collection, storage, and DNA extraction methods, though many lacked details on time from collection to freezing. Of the 22 studies using 16S rRNA sequencing, 21 reported the taxonomic reference database and 17 specified the database version. Among these 21 studies, nine employed amplicon sequence variants (ASVs) for taxonomic classification while 13 used operational taxonomic units (OTUs). Raw sequencing data were publicly available for 16 studies, while ten studies did not report accession numbers or make their data publicly accessible. All studies reported sequencing methods and bioinformatic pipelines; further details are provided in the supplementary materials (Table S2).

SEP was measured by various individual- and neighbourhood-level indicators, with 14 studies assessing multiple SEP measures. The most common individual-level SEP measures were education and income. Education was typically assessed as the highest qualification achieved or years of schooling, while income was reported at the household level in seven studies and individual level in three.^1^^,^^26^^,^^27^ Three LMIC studies used asset-based SEP indices,^8^^,^^30^^,^^38^ including household items, water access, and flooring materials, though weighting methods were not reported. Seven studies assessed neighbourhood-level SEP (Table 2). SEP measurement varied by life stage, with infant and child studies predominantly using parental education, household income, and school-level measures, while adult studies primarily employed individual income, educational attainment, neighbourhood deprivation indices, and occupational measures such as the Occupational Socioeconomic Index.

**Table 2.** Summary of the neighbourhood-level SEP exposures examined in the reviewed literature.

Neighbourhood-level SEP	Description
Neighbourhood income^1^	Average neighbourhood income using Statistics Netherlands (2015).
IMD (Index of Multiple Deprivation)^6^	Measure of relative deprivation in England for small areas, combining 37 indicators grouped into seven domains: income, employment, education, health, crime, barriers to housing and services, and living environment.
SDI (Socioeconomic Deprivation Index)^31^	Composite measure of area-level deprivation based on seven measures from the American Community Survey (ACS): poverty, education, single-parent households, rented and overcrowded housing, car access, and unemployment among adults under 65.
NZDep2013 (New Zealand Deprivation Index 2013)^22^	Deprivation score for each meshblock, a geographic unit defined by Statistics New Zealand with a median population of approximately 81 people in 2013. Combines nine variables across eight domains: internet access, income, employment, education, home ownership, family structure, overcrowding, and car access.
EHI (Economic Hardship Index)^40^	Measure of economic conditions using six socioeconomic factors from the 2014 5-Year ACS data. Includes: unemployment, dependency, educational attainment, per capita income, crowded housing, and poverty.
Neighbourhood SEP score^36^	Constructed by the author using neighbourhood median household income, educational attainment (percentage of adults over 25 with a high school diploma), employment (percentage of adults seeking work), and median owner-occupied home value.
Neighbourhood SEP score^21^	Constructed by the author using the same variables as Miller et al.^36^ but includes additional variables: percentage of households receiving public assistance, households below the federal poverty threshold, and housing conditions (median owner-occupied home value and percentage of vacant households).

Quality assessment classified five studies as good, 17 as fair, and four as poor. Poor-rated studies lacked clearly stated research questions, defined populations, valid SEP measures, and any confounder adjustment. Fair-rated studies met most quality criteria but inconsistently adjusted for key confounders. Good-rated studies demonstrated clear population definitions, larger samples (700–2,000 participants), valid and diverse SEP measures, robust methods, and comprehensive confounder adjustment.

Across studies, results were often framed primarily in terms of statistical significance, sometimes without providing exact *p*-values or effect size estimates. Significance thresholds differed across studies, with most using FDR-adjusted q < 0.05, some q < 0.01, and others using unadjusted *p* < 0.05.

**Figure 2.**
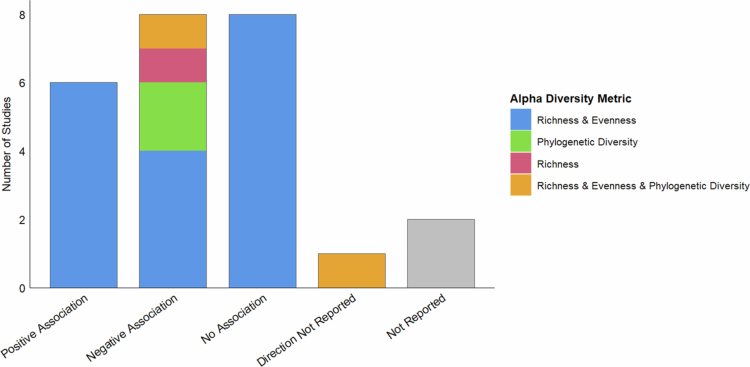
Summary of *α*-diversity results coloured by the type of metric used. Blue: richness and evenness; green: phylogenetic diversity; red: richness only; yellow: all metric types. Levin et al.^34^ from a HIC (USA) reported a significant association, but the direction was unspecified; Galley et al.^24^ was excluded from Figure 2 because the observed association between SEP and *α*-diversity was dependent on obesity status.

In the reviewed literature, 24 studies examined *α*-diversity, with a variety of metrics being used. Different *α*-diversity measures capture distinct aspects of diversity, such as richness, evenness, or phylogenetic relationships. 15 studies reported a significant association between SEP and GM *α*-diversity, however the direction of association varied (Figure 2), with six studies reporting a positive association and eight reporting a negative association.

Negative associations—where disadvantaged SEP linked to higher *α*-diversity—were mostly from LMICs.^8^^,^^19^^,^^30^^,^^33^^,^^38^ Although conducted in Israel, a HIC, Lapidot et al.^33^ focused on the Arab minority, who are socioeconomically disadvantaged compared to the Jewish majority. Other studies from HICs that found negative associations included two US ethnically diverse cohorts.^28^^,^^31^ and one Australian pre-term infant study.^22^ Positive associations—where disadvantaged SEP linked to lower *α*-diversity—were only found in HICs, mainly the US and Europe.^1^^,^^6^^,^^29^^,^^36^^,^^39^^,^^40^ Most involved predominantly participants identifying as “White”; only one had a more ethnically diverse but extremely small sample.^36^

Some studies reported specific SEP–α-diversity associations. Galley et al.^24^ found that in high-income households, children of obese mothers had higher GM *α*-diversity than those of non-obese mothers, however this was not observed in low-income households. Kwak et al.^31^ observed a positive association between educational attainment and *α*-diversity, but no association with neighbourhood income or deprivation. Mulder et al.^39^ found that among multiple early life stress domains, only socioeconomic stress and low maternal education were associated with GM diversity, suggesting that SEP may significantly influence the early-life microbiome.

**Figure 3.**
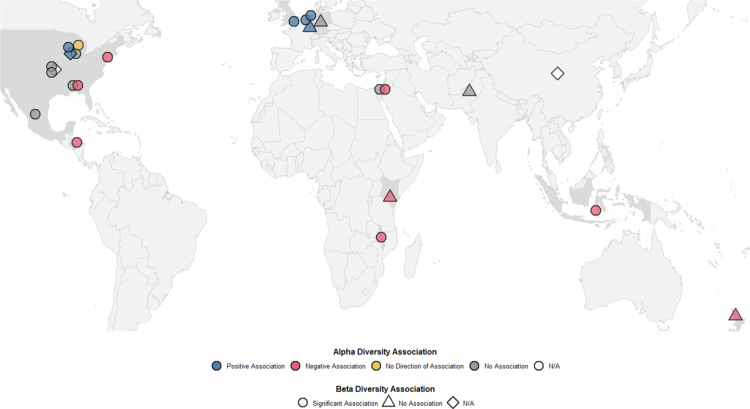
Geographic distribution of studies investigating the association between SEP and GM diversity. Galley et al.^24^ was excluded from Figure 3 because the observed associations between SEP and diversity were dependent on obesity status. Symbol colours represent *α*-diversity associations (blue: significantly positive; red: significantly negative; yellow: significant but unspecified direction; grey: non-significant; white: not examined). Symbol shapes indicate *β*-diversity associations (circle: significant; triangle: non-significant; diamond: not examined). Grey-shaded countries had at least one study reporting a significant association. Symbols are placed at country centroids when specific locations were not provided (e.g., USA, China).

Across the reviewed literature, 23 studies examined *β*-diversity, with a range of metrics being used. The most commonly used measure was Bray-Curtis dissimilarity, however many studies employed multiple measures. 17 studies found significant associations between SEP and *β*-diversity, six found no such association, and three did not assess *β*-diversity (Figure 3). Two studies reported specific SEP–β-diversity correlations. Zuniga-Chaves et al.^40^ found an association only when comparing the top 15% most disadvantaged (85th percentile EHI) to the rest of the sample, with no difference at the 50th percentile split suggesting non-linear associations. Similarly to *α*-diversity, Galley et al.^24^ observed that *β*-diversity differed significantly between children of obese and non-obese mothers only within the high-income group.

19 studies reported differences in taxonomic composition, identifying 358 unique taxa significantly associated with SEP, predominantly at the genus level. Of these, 61 taxa showed consistent associations with SEP across two or more studies. Associations spanned five phyla: *Bacteroidetes, Firmicutes, Actinobacteria*, *Proteobacteria*, and *Verrucomicrobia*, with *Firmicutes* being the most frequently reported. At the phylum level, associations with SEP were typically evenly distributed between positive and negative effects across both HICs and LMICs (Figure 3A, supplementary materials).

**Figure 4.**
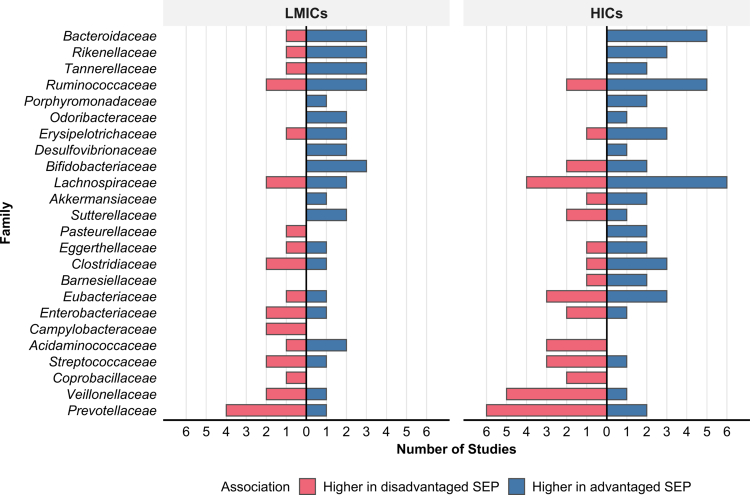
Family-level taxonomic associations with SEP across HICs and LMICs. Blue: higher abundance in advantaged SEP; red: higher in disadvantaged SEP. Bars represent the number of studies reporting associations for each taxon. Only taxa with ≥2 studies reporting the same direction of association (positive or negative) in either population are shown.

At the family level, *Lachnospiraceae, Ruminococcaceae*, and *Prevotellaceae* were most frequently associated with SEP (Figure 4). *Prevotellaceae* showed predominantly negative associations in both HICs and LMICs, while *Ruminococcaceae* was predominantly positively associated in HICs with mixed patterns in LMICs. *Lachnospiraceae* exhibited mixed associations in both populations, though slightly favouring positive associations in HICs. Several families exhibited population-specific patterns: *Bifidobacteriaceae* showed mixed associations in HICs but exclusively positive associations in LMICs, while *Acidaminococcaceae* was negatively associated in HICs but more positively in LMICs. *Bacteroidaceae* showed consistent positive associations across both populations, with only a single negative association reported in LMICs.

**Figure 5.**
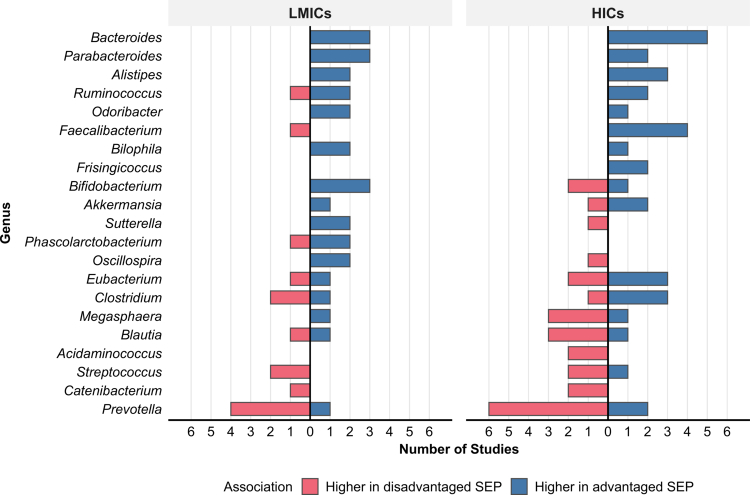
Genus-level taxonomic associations with SEP across HICs and LMICs. Blue: higher abundance in advantaged SEP; red: higher in disadvantaged SEP. Bars represent the number of studies reporting associations for each taxon. Only taxa with ≥2 studies reporting the same direction of association (positive or negative) in either population are shown.

21 genera were associated with SEP in two or more studies, with *Bacteroides* and *Prevotella* most frequently reported (eight and thirteen studies, respectively; Figure 5). *Bacteroides* showed exclusively positive associations across both HICs and LMICs, indicating a consistent pattern for this genus, while *Prevotella* was predominantly associated with disadvantaged SEP in both settings. Several genera exhibited population-specific patterns. *Bifidobacterium* was exclusively positively associated with advantaged SEP in LMICs but showed predominantly negative associations in HICs (two of three studies). *Faecalibacterium* was consistently positively associated in HICs (4 studies), with only one LMIC study reporting a negative association. Some taxa were population-specific: *Frisingicoccus* and *Acidaminococcus* were reported only in HICs, whereas *Phascolarctobacterium* appeared exclusively in LMICs.

Nine studies reported associations between SEP and species-level taxa,^1^^,^^6^^,^^8^^,^^23^^,^^27^^,^^31^^,^^33^^,^^34^^,^^40^ with *Prevotella copri* most frequently identified, followed by *Faecalibacterium prausnitzii* and *Bacteroides fragilis*. *P*. *copri* was consistently associated with disadvantaged SEP across five studies in both HICs and LMICs. In contrast, B. *fragilis* was associated with advantaged SEP in LMICs (three studies), while F. *prausnitzii* showed mixed patterns, with one association with disadvantaged SEP in LMICs and exclusively positive associations in HICs (three studies). Only one study in this review examined strain-level differences by SEP in an LMIC, finding that wealthier individuals harboured distinct strains of *Eubacterium rectale* compared to those with disadvantaged SEP.^8^ SEP was the strongest predictor of GM variation among the factors examined—highlighting the importance of investigating strain-level taxonomic differences by SEP, especially given that strains within the same species can differ in functional activity.

**Figure 6.**
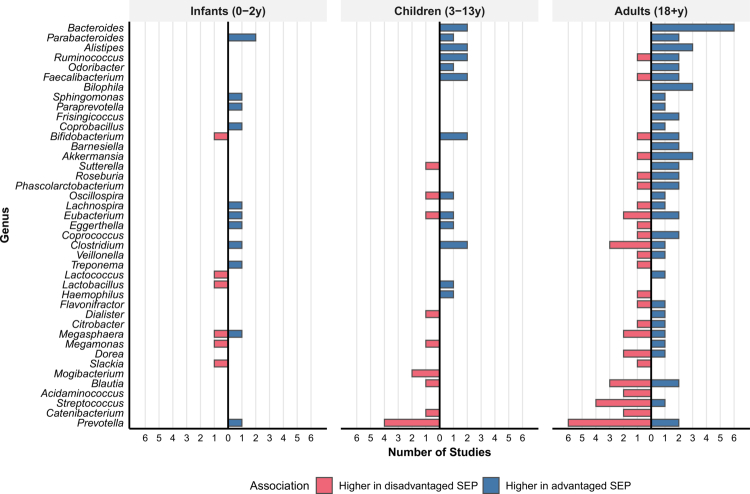
Genus-level taxonomic associations with SEP across life stages. All taxa associated with SEP in any age group are displayed. Blue: higher abundance in advantaged SEP; red: higher in disadvantaged SEP. Bars represent the number of studies reporting associations for each taxon. The infant panel is predominantly represented by findings from a single HIC study,^34^ which reported multiple taxonomic associations, whereas the other two infant studies reported fewer associations.

Cross-life stage comparisons are limited by the predominance of studies conducted in adults and in HICs, as well as by the scarcity of replicated findings in younger age groups; only five taxa in children and one taxon in infants showed consistent directional associations across multiple studies. Despite these limitations, age-stratified analyses reinforce the overall findings of higher *Prevotella* abundance in disadvantaged SEP groups and higher *Bacteroides* (and the related *Parabacteroides*) abundance in advantaged SEP groups across the life stages (Figure 6). However, we observe some life stage-specific patterns. *Bifidobacterium* showed an association with disadvantaged SEP in infants^22^ but predominantly with advantaged SEP in children and adults.^8^^,^^19^^,^^27^^,^^33^ Similarly, *Clostridium* was associated with advantaged SEP in infants and children,^32-34^ whereas in adult studies, three studies reported associations with disadvantaged SEP.^1^^,^^8^^,^^27^ In adults, strong SEP associations emerge across multiple taxa beyond *Prevotella* and *Bacteroides*, including *Blautia, Streptococcus, Clostridium, Eubacterium*, and *Akkermansia*. Furthermore, age-stratified analysis reveals that significantly positive SEP-*α*-diversity associations were predominantly found in adult studies (supplementary Figure 5), whereas significantly negative associations were observed consistently across all life stages. Additional figures illustrating SEP-microbiome associations across life stages and at all taxonomic levels are provided in the supplementary materials (Figures 2A−4E).

Table 3 summarises the functional and health implications of the most frequently reported SEP-associated taxa, with SCFA production emerging as a predominant function. SCFAs, produced by anaerobic fermentation of indigestible polysaccharides like fibre and starch, exhibit anti-inflammatory and antimicrobial effects, support gut integrity, and play a major role in the gut-brain axis.^46^ Acetate, propionate, and butyrate—the primary SCFAs produced by GM^41^—play key roles in health, with *Bifidobacterium spp*., *Faecalibacterium prausnitzii*, and *Bacteroides fragilis* identified as main producers,^47^ all found to be associated with SEP in this review. 

Eight studies conducted functional analyses, using tools such as PICRUSt2 for 16S rRNA data and HUMAnN or ShotMAP for shotgun metagenomics; six reported associations between SEP and metabolic pathways.^1^^,^^8^^,^^23^^,^^31^^,^^33^^,^^39^ Collectively, these studies identified 182 SEP-associated pathways, with five showing consistent associations across more than one study (Table 4), illustrating how socioeconomic disparities may influence metabolic processes relevant to host health. Three studies examined SEP-functional pathway associations in children^23^^,^^33^^,^^39^ and three in adults.^1^^,^^8^^,^^31^ In children, no pathways showed replicated associations across studies. Across all life stages, most identified pathways were associated with advantaged SEP, with only one exception: a TCA cycle pathway (incomplete reductive citrate cycle, acetyl-CoA to oxoglutarate) was associated with disadvantaged SEP in two studies.

**Table 3.** Frequently reported SEP-associated microbial taxa (genera [g] and species [s]), including direction of association, key functions, and known links to health and disease.

Taxon	Association with SEP	Functional implications	Associations with health and disease	References
*Bacteroides (g)* *B. ovatus (s)* *B. uniformis (s)* *B. fragilis (s)*	Advantaged SEP in HICs and LMICs	Core member of GM; produces acetate, propionate, butyrate, and succinate; involved in protein and glycan metabolism; anti-inflammatory effects; diverse antimicrobial resistance mechanisms.	Reduced in patients with CD, colitis, IBD.	[48,49]
*Prevotella (g)* *P. copri (s)*	Predominantly disadvantaged SEP in HICs and LMICs	Metabolises carbohydrates and mucin; produces propionate; *P. copri* is the most dominant species in the genus.	Associated with inflammatory conditions; high abundance linked to rheumatoid arthritis; reduced in obesity.	[50]
*Eubacterium (g)*	Advantaged SEP in HICs, Disadvantaged SEP in LMICs	Produces butyrate and propionate; species can carry out bile acid and cholesterol transformations in the gut.	Reduced in IBD; associated with obesity; considered a target for microbial therapeutics.	[51]
*Alistipes (g)*	Advantaged SEP in LMICs	Produces acetate; involved in inflammation modulation and potential role in the gut-brain axis.	Protective in liver fibrosis, colitis, and cardiovascular disease; pathogenic in CC and depression.	[52]
*Parabacteroides (g)*	Advantaged SEP in HICs	Produces acetate, propionate, and butyrate; wide carbohydrate utilisation abilities; *P. distasonis* exhibits notable antibiotic resistance.	Found to be depleted in obesity, metabolic syndrome, and IBD.	[53]
*Bifidobacterium (g)*	Advantaged SEP in LMICs	Produces acetate and lactic acid; one of the earliest gut colonisers; involved in metabolic regulation, immune modulation, and pathogen protection.	Regarded as a key bacterium with health-promoting effects throughout life; certain strains are established probiotics.	[54]
*Faecalibacterium (g) Faecalibacterium prausnitzii (s)*	Predominantly advantaged SEP in HICs and LMICs	Produces butyrate and anti-inflammatory molecules (e.g., shikimic and salicylic acids); supports metabolic and immune regulation, and colon protection.	Depleted in various gut diseases; negatively associated with pathogenesis of CD, IBD, and prostate cancer; potential probiotic candidate.	[55]
*Akkermansia (g) Akkermansia muciniphila (s)*	Predominantly advantaged SEP in HICs	Mucin degradation; production of acetate and propionate; maintains intestinal mucus integrity.	Associated with reduced inflammation; protective against metabolic, neurological, and infectious diseases; reduced abundance in obesity, type 2 diabetes, IBD.	[56]
*Clostridium (g)*	Mixed associations in HICs and predominantly advantaged SEP in LMICs	Core microbiome genus; produces butyrate; probiotic role via supporting epithelial energy, gut barrier integrity, and immune interaction. Genus contains both beneficial (C. *butyricum*) and pathogenic species (C. *difficile*).	Linked to obesity; decreased in CD; C. *difficile* can cause infections and gut dysbiosis.	[57–59]

HICs = high-income countries; LMICs = low- and middle-income countries; CD = Crohn’s disease; IBD = inflammatory bowel disease; CC = colorectal cancer; IBS = irritable bowel syndrome.

**Table 4.** Summary of microbial pathways associated with SEP, including direction of association, pathway functions, and potential health implications.

Microbial pathway	Association with SEP	Function and association with health	Potential health implications	References
TCA cycle IV (2-oxoglutarate decarboxylase)	Disadvantaged SEP in HICs	Oxidises acetyl-CoA from carbs, fats, and proteins to produce NADH and FADH₂ for ATP; this variant converts 2-oxoglutarate to succinate through succinate semialdehyde, bypassing ATP synthesis, maintaining biosynthetic precursor and electron carrier production even at high ATP levels.	May reflect dietary patterns linked to disadvantaged SEP in HICs and less efficient energy metabolism.	-
Fucose degradation	Advantaged SEP in HIC and LMIC	Fucose, a key gut sugar, is released by bacterial fucoidases (e.g., *Bacteroides* spp.) and metabolised into SCFAs used by microbes; supports host–microbiome interactions.	May indicate increased SCFA production, reflecting a diverse, health-promoting microbiota.	[41]
Polyamine biosynthesis I	Advantaged SEP in HIC and LMIC	Polyamines regulate gene expression, cell growth, stress response, and immune modulation, including reducing inflammation and supporting gut barrier; probiotics can raise polyamine levels, linked to increased lifespan in mice.	May indicate a health-beneficial microbiota, potentially linked to probiotic-rich diets.	[42]
Purine ribonucleosides degradation	Advantaged SEP in HIC and LMIC	Breaks down purine nucleosides into uric acid; gut bacteria use purines as carbon/energy sources; higher abundance of purine-degrading bacteria linked to lower circulating uric acid; elevated uric acid is linked to noncommunicable diseases such as atherosclerosis.	May suggest protective effects via reduced uric acid levels.	[43]
L-lysine biosynthesis I and II	Advantaged SEP in 2 studies (HIC and LMIC); disadvantaged SEP in 1 study (HIC)	Produces essential amino acid L-lysine from aspartate, important for growth, bone development, and calcium absorption; poly-L-lysine has been found to exert anti-inflammatory effects in the gut.	May support anti-inflammatory effects.	[44,45]

HICs = high-income countries; LMICs = low- and middle-income countries; SCFAs = short-chain fatty acids.

## Discussion

This systematic review provides a comprehensive overview of studies examining the relationship between GM and individual- and neighbourhood-level SEP across diverse populations and ages. Individuals from disadvantaged SEP groups consistently exhibited distinct microbiomes compared to those from advantaged backgrounds as observed by significant associations between SEP and *β*-diversity. Studies reporting no association had small sample sizes (<200), suggesting insufficient statistical power to detect such differences and underscoring the need for larger cohorts to examine SEP-related microbiome variation.

### Alpha diversity: Geographic variation and the epidemiological transition

Defining a “healthy” GM is challenging due to substantial intra-individual variability; however, high microbial diversity combined with the presence and abundance of specific beneficial taxa and functional pathways are generally seen as indicators of good gut health.^60^ A-diversity showed contrasting patterns by geography: disadvantaged SEP was associated with increased diversity in LMICs but decreased diversity in HICs (Figure 3). These opposing findings highlight the complexity of SEP–α-diversity relationships, which are influenced by diet, BMI, ethnicity, and geography—factors that many studies in this review did not fully account for.

Geography notably shapes gut microbial composition, with consistent patterns observed in HICs^1^ but distinct profiles in LMICs and populations undergoing urbanisation.^60^ In HICs, the SEP–α-diversity relationship reinforces the link between advantaged SEP and better health outcomes,^6^ with higher *α*-diversity associated with better health and viewed as a marker of a more stable, resilient gut ecosystem. Lower *α*-diversity is generally recognised as a hallmark of dysbiosis, a disrupted microbiome state often linked to disease development.^61^ However, this relationship between SEP and *α*-diversity varied in LMICs, where disadvantaged SEP was associated with higher *α*-diversity. Most microbiome research focuses on HICs, with limited representation of LMICs and ethnic minorities, as evidenced by this review.

This review’s *α*-diversity findings align with the epidemiological transition, which describes the shift from mortality being predominantly caused by infectious diseases to chronic, non-communicable conditions as societies undergo economic development.^62^ From this perspective, health disparities arise as lower-income and ethnic minority groups often undergo this shift more slowly than advantaged SEP groups. In developing countries, industrialisation drives dietary changes—such as higher intake of processed, high-fat foods—and reduced physical activity, leading to increased BMI and obesity, which are linked to lower gut *α*-diversity.^63^ Non-industrialised populations show higher gut microbial diversity than industrialised populations, suggesting industrialisation is driving global microbial loss.^60^

The opposing SEP–diversity associations observed in HICs and LMICs highlight that SEP operates through context-specific mechanisms. This challenges the notion of universal microbiome-based health interventions and suggests that strategies to address SEP-related microbiome disparities must be tailored to population-specific contexts.

### Life-stage patterns in SEP–microbiome associations

Beyond geography, country income classification, and ethnicity, age is a critical moderator of SEP–microbiome associations, given substantial life-course changes in the gut microbiome and age-related differences in how SEP is measured. SEP also operates through distinct mechanisms across the life course, shaping delivery mode, feeding practices, and environmental exposures in early life, and reflecting cumulative exposures alongside current living conditions in adulthood. Whether early-life SEP establishes enduring microbiome trajectories or can be modified by later-life SEP remains unclear, highlighting the need for longitudinal life-course studies.

Some studies observed age-dependent associations for specific taxa. *Clostridium*, for instance, was predominantly associated with advantaged SEP in infant and child studies but with disadvantaged SEP in adult studies. Interpretation at the genus level is challenging because *Clostridium* encompasses both beneficial species and pathogens,^57^ and the species linked to SEP likely differ across age groups. Associations with advantaged SEP in early life may reflect beneficial, commensal species promoted by breastfeeding and dietary diversity, whereas associations with disadvantaged SEP in adulthood may indicate opportunistic species favoured by poor diet quality, chronic stress, and adverse environmental conditions. This finding also highlights the limitations of genus-level taxonomic resolution for understanding SEP–microbiome relationships. Without species- or strain-level identification, such as through shotgun metagenomics, it is unclear whether observed findings reflect biologically meaningful shifts or simply changes in the species across life stages. *Bifidobacterium* showed the opposite pattern, with one infant study reporting higher abundance in individuals with disadvantaged SEP. This finding is unexpected, as many *Bifidobacterium* species are established early in life through vaginal delivery and breastfeeding.^11^ The predominant association of *Bifidobacterium* with advantaged SEP in child and adult studies may reflect greater dietary diversity and access to prebiotic foods; however, inconsistency within age groups prevents conclusive interpretation.

### Pathways linking SEP to the microbiome and health

SEP and health are theoretically linked through material, psychosocial, and behavioural pathways.^3^^,^^7^ Material factors encompass environmental conditions such as sanitation, crowding, water access, animal exposure, and pollution; psychosocial factors include chronic stress, income insecurity, social support, and discrimination; and behavioural factors include diet, breastfeeding, physical activity, smoking, alcohol use, and healthcare access. Applying this framework to SEP–microbiome associations helps contextualise our findings and illustrates how these pathways shape microbiome composition across settings and life stages, with downstream effects on immune, metabolic, and inflammatory processes.

Disadvantaged SEP differs greatly between HICs and LMICs; in LMICs, it often involves poor access to clean water, inadequate sanitation, and increased animal exposure, leading to greater microbial and parasitic contact that may elevate *α*-diversity.^33^ Household crowding, an indicator of disadvantage and stress, is linked to higher morbidity and mortality.^64^ It increases interpersonal contact, potentially promoting microbial exchange and raising *α*-diversity through both beneficial and harmful bacteria. Zuniga-Chaves et al.^40^ reported that greater economic hardship was associated with increased colonisation and diversity of multidrug-resistant organisms, potentially reflecting reduced microbiome resilience in disadvantaged populations. However, gaps remain in understanding how household crowding influences horizontal microbiota transmission and whether associations are confounded by antibiotic use, diet, or healthcare access.

In HICs, advantaged SEP groups typically have greater access to nutritious, high-fibre, and fermented foods linked to greater microbial diversity, while disadvantaged groups face food insecurity and consume cheaper, high-fat, low-fibre diets linked to lower microbial diversity.^3^^,^^65^ Dietary fibre directly provides substrate for SCFA-producing bacteria; its absence deprives beneficial commensals of energy sources. Economic constraints drive consumption of inexpensive, calorie-dense processed foods that are nutrient-poor and fibre-deficient. Overall, disadvantaged SEP in HICs is associated with nutritional trade-offs, chronic stress, altered neonatal health (e.g., lower breastfeeding, higher caesarean rates), and increased antibiotic use—along with various other factors—all contributing to reduced microbiota diversity, increased pro-inflammatory species, and poorer metabolic health. In LMICs, dietary patterns show opposite SEP gradients: westernised, processed diets are more accessible to wealthier individuals, while less wealthy groups continue to consume traditional, fibre-rich, agrarian diets, potentially contributing to their higher *α*-diversity.^66^ However, patterns in LMICs are less well established due to limited data and ongoing industrialisation, contributing to considerable variation between populations in LMICs.^67^

### Ethnicity as a modifier of SEP–microbiome associations

Alongside urbanisation, ethnicity plays a role in shaping the link between SEP and the GM. Ethnic background shapes culture, which in turn influences diet, lifestyle, and childcare practices—key factors driving GM variation.^68^ Comparisons between HICs and LMICs may reflect ethnic as well as geographical differences.

Studies in HICs showing positive SEP–α-diversity links mostly involved European participants, while those reporting negative associations had more ethnically diverse cohorts. In the US, Kwak et al.^31^ found that participants identifying as “Black”, “Hispanic”, and “foreign-born” had more disadvantaged SEP compared to “White” or “US-born individuals”. They and others^28^^,^^33^ also observed higher *α*-diversity—specifically Faith’s Phylogenetic Diversity (PD)—among individuals with disadvantaged SEP, with significant differences across ethnic groups. However, no other studies in this review examined Faith’s PD in relation to SEP, and those reporting positive associations used different metrics, limiting conclusions. These findings suggest ethnic minorities in HICs may retain dietary and cultural practices supporting microbial diversity despite socioeconomic disadvantage. Overall, confounding from the collinearity between ethnicity and SEP makes it challenging to isolate the specific effects of SEP on the GM.

### Microbiome and health

Having discussed how SEP shapes microbiome composition through multiple pathways, we now describe how these microbiome differences may affect health through taxonomic profiles, functional capabilities, and host interactions.

### Taxonomic associations and enterosignatures

Beyond diversity, several taxa and metabolic pathways showed consistent associations with SEP. Distinct bacterial enterosignatures have been characterised: *Bacteroides*-dominated profiles are more common in Western populations, while *Prevotella*-dominated profiles predominate in non-Western groups.^69^ These patterns primarily reflect dietary differences—Western diets high in animal protein and fat favour *Bacteroides*, whereas plant-based diets rich in complex carbohydrates and fibre promote *Prevotella.*^70^ Migration studies demonstrate the microbiome's rapid responsiveness to dietary change: Southeast Asians moving to the USA show replacement of *Prevotella* strains providing plant fibre-degrading enzymes with *Bacteroides* species.^10^ This plasticity suggests microbiome-targeted interventions could potentially attenuate SEP-associated microbiome differences through dietary and environmental modifications.

We identified associations between advantaged SEP and higher *Bacteroides* abundance, and disadvantaged SEP and higher *Prevotella*, across HICs and LMICs and age groups. However, the functional and health implications of these enterosignatures likely vary by context. In HICs, *Bacteroides* enrichment with advantaged SEP likely reflects access to diverse, animal-protein-rich diets. *Prevotella* enrichment with disadvantaged SEP is more ambiguous: it may reflect retained traditional fibre-rich diets in ethnic minorities, greater reliance on plant-based staples (legumes, grains), or distinct *Prevotella* species/strains with different functional capabilities.^71^ In LMICs, *Prevotella* enrichment more likely reflects traditional, fibre-rich diets. Without detailed dietary data and species-level resolution, rarely available in reviewed studies, *Prevotella* enrichment with disadvantaged SEP cannot be interpreted as beneficial or detrimental.

### Functional pathways: SCFAs and metabolic health

The GM exhibits considerable functional redundancy: that is different taxonomic compositions can perform similar metabolic functions, while similar taxonomic profiles may differ substantially in functional output.^72^ This underscores the importance of examining microbial function alongside taxonomy when interpreting SEP–microbiome relationships.

SCFA production emerged as the predominant function among SEP-associated taxa. SCFAs regulate glucose homeostasis, lipid metabolism, immune tolerance, blood-brain barrier integrity, and neural function,^46^^,^^47^ processes directly implicated in the chronic diseases that show strong SEP gradients such as obesity, diabetes, and cardiovascular disease.^5^ If reduced SCFA production contributes to SEP–health disparities, the microbiome may represent a modifiable intervention target. However, critical evidence gaps remain: most studies examined only taxonomic composition rather than SCFA concentrations or functional profiles.

The GM produces neurotransmitters (GABA, serotonin, dopamine) and their precursors,^73^ contributing to gut-brain axis communication. Several SEP-associated taxa identified in this review—including *Bifidobacterium*, *Parabacteroides*, and *Bacteroides fragilis*—are established GABA producers,^73^ raising the possibility that socioeconomic microbiome differences could contribute to mental health disparities. However, no studies have directly linked SEP-associated microbiome changes to neurotransmitter levels or mental health outcomes.

The microbiome also influences health through immune regulation, gut barrier integrity, and metabolic pathways relevant to SEP-related disparities. Early-life microbial exposure shapes immune development and tolerance;^11^ disrupted colonisation—from lower breastfeeding rates, higher caesarean delivery rates, and greater antibiotic exposure in disadvantaged populations—may impair immune development and increase the risk of allergic diseases and asthma, which exhibit SEP gradients.^74^ The microbiome maintains barrier integrity through metabolite production and tight junction regulation; barrier dysfunction allows bacterial products (e.g., lipopolysaccharide) to translocate, driving chronic low-grade inflammation implicated in obesity, diabetes, and cardiovascular disease.^75^ Additionally, microbial regulation of bile acid metabolism, vitamin synthesis, and nutrient bioavailability further links the microbiome to metabolic health. Although few studies directly examined associations between SEP and GM functional pathways, only five pathways were consistently associated across two studies, limiting definitive interpretation. These diverse pathways through which the microbiome influences health establish it as a critical mediator linking SEP to health outcomes, offering a potentially modifiable target for interventions aimed at reducing health inequities.

## Heterogeneity, limitations, and recommendations

This review identifies key knowledge gaps and limitations to guide future research. Most studies were cross-sectional, limiting the ability to establish temporality and assess changes in microbiome composition over time. None of the studies conducted power calculations, raising concerns about whether they were sufficiently powered to detect associations. Sample collection and processing protocols also varied widely, with some studies failing to report key details such as time between collection and freezing. Furthermore, some studies had socially homogeneous, generally advantaged samples with narrow SEP ranges,^6^ limiting capture of full socioeconomic variation.

SEP measurement varied substantially across studies, reflecting both developmental stage and geographic context, precluding meta-analysis. LMIC studies typically used asset-based indices, whereas HIC studies relied on income, education, or occupation. Infant and child studies predominantly used household-level or parental SEP measures, whereas adult studies employed individual-level indicators. Some combined multiple indicators into cumulative scores; others analysed them separately, complicating comparisons. These differences reflect a deeper challenge: SEP indicators are not directly comparable across life stages due to both conceptual and measurement differences. Moreover, SEP is dynamic, not captured by measurement at a single time point—it changes over time and may exert effects that are cumulative, time-sensitive, or life-stage-specific. Early-life SEP may exert critical-period influences on microbiome development, whereas adult SEP more often reflects accumulated exposures. Age-specific confounding further complicates cross-age comparisons. Evidence on SEP–microbiome relationships in infancy remains sparse, despite the “first 1000 days” representing a critical developmental period during which SEP may exert particularly influential effects.

Study populations varied widely, ranging from healthy to diseased participants and spanning infancy to adulthood. Exclusion criteria differed, including inconsistent handling of recent antibiotic use. Inconsistent adjustment for key covariates may have biased findings or caused relevant studies to be missed. Some studies did not report effect sizes and variance measures necessary for meta-analysis, providing only *p*-values or qualitative descriptions of significant associations.

To improve comparability across diverse socioeconomic and geographic contexts, future studies should adopt standardised SEP measurements that account for geographic context (e.g., asset-based indices in LMICs; income or education in HICs) and life stage (household-level indicators for children; individual-level measures for adults), while clearly reporting which indicators were used and how they were operationalised. Analytical rigour would be enhanced by consistently collecting and adjusting for core confounders—including age, sex, BMI, antibiotic use, health behaviours, and early-life exposures such as delivery mode and breastfeeding—and reporting effect sizes, confidence intervals, and variance measures rather than *p*-values alone. Transparent reporting of recruitment strategies, including response rates and population characteristics, is also needed.

Substantial methodological heterogeneity in microbiome characterisation also prevented meta-analysis. Among 16S rRNA studies, considerable variation existed in hypervariable regions sequenced, sequence processing methods (OTUs vs. ASVs), bioinformatic pipelines, and reference databases—differences known to affect taxonomic identification and abundance estimates. Some recent studies continued using OTU-based clustering^6^ despite the availability of ASV methods, which provide higher taxonomic resolution and greater reproducibility by preserving exact sequence variants rather than clustering reads at arbitrary similarity thresholds.^76^ While OTU use may be justified in earlier studies predating ASV standardisation, its continued use in recent studies represents a missed opportunity for improved resolution. Additionally, five studies relied on the outdated GreenGenes (v13.8) database from 2013,^9^^,^^30^^,^^32^^,^^33^^,^^37^ and two used outdated SILVA versions despite more recent releases being available.^26^^,^^29^

Beyond these methodological choices, inherent limitations of 16S rRNA sequencing constrain interpretation of SEP–microbiome associations, including limited taxonomic resolution, susceptibility to primer bias, and inability to assess functional potential.^77^ Shotgun metagenomic sequencing overcomes these limitations by providing species- and strain-level resolution, functional profiling, and detection of multiple microbial domains.^78^ Indeed, the fungal microbiome (mycobiome) is increasingly recognised as an important determinant of health and disease,^79^ yet to our knowledge, its relationship with SEP remains unexplored.

Data quality and reproducibility would be strengthened through detailed reporting of sample collection protocols (including time to freezing), DNA extraction, sequencing parameters, and bioinformatic pipelines, ideally following established guidelines such as STORMS.^80^ Depositing raw sequencing data in public repositories would further enhance cross-study comparability and facilitate meta-analyses.

**Figure 7.**
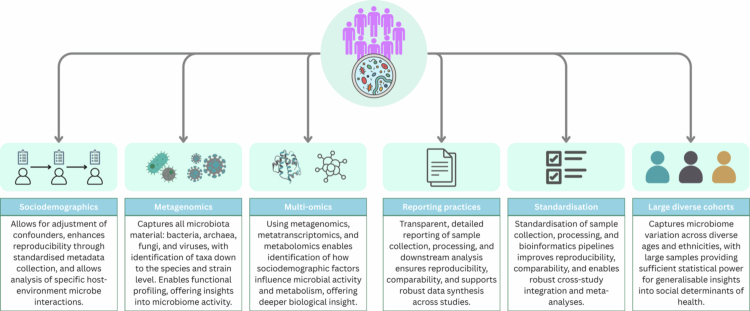
Recommendations for future GM social epidemiology research: ensure collection of sociodemographic data and key covariates; adoption of metagenomic sequencing for greater taxonomic resolution; integration of multi-omics approaches to capture microbial activity and metabolism; improved transparency in methods and analysis to support reproducibility and cross-study comparability; increased standardisation across microbiome studies; and the use of large, diverse cohorts to ensure adequate statistical power and generalisable findings. Created with Canva.

As the field evolves, best practices continue to shift, complicating fixed “gold-standard” guidelines; nevertheless, clearer guidance and improved reporting remain essential (Figure 7). Future studies should prioritise: (1) implementing life-course study designs that capture SEP exposure across multiple developmental stages; (2) conducting power calculations and recruiting diverse cohorts with broad SEP ranges; (3) collecting and adjusting for key covariates consistently; (4) adopting metagenomics or multi-omics approaches for improved taxonomic and functional resolution; (5) comprehensive reporting following established guidelines such as STORMS; and (6) using up-to-date databases like SILVA for 16S rRNA sequencing.

Despite these limitations, the findings demonstrate that SEP influences GM composition, likely through different pathways across geographic, ethnic, and developmental contexts. In HICs, disadvantaged SEP associates with reduced SCFA-producing bacteria and lower *α*-diversity, whereas in LMICs, disadvantaged populations typically exhibit higher *α*-diversity. These patterns position the microbiome as a potential biological pathway partially mediating SEP–health disparities and a promising intervention target. Given its plasticity to dietary and environmental factors, interventions could operate at multiple levels: targeted delivery of beneficial microbes or metabolites (e.g., SCFA-producers), dietary interventions promoting fibre-rich and minimally processed foods, early-life interventions that promote breastfeeding and healthy colonisation, and policy measures addressing food insecurity, maternal health support, and sanitation in disadvantaged communities. Such interventions may help reduce health inequities, provided future studies establish causal relationships and identify microbiome changes with meaningful health impacts.

## Disclosure of potential conflicts of interest

We declare no competing interests.

## Supplementary Material

Supplementary_Materials_Figures.docx Supplementary_Materials_Figures.docx

Supplementary_Materials_Tables.xlsx Supplementary_Materials_Tables.xlsx

## Data sharing

The authors confirm that the data supporting the findings of this study are available within the article and its supplementary materials.
