# Supplementary material for: Socioeconomic position and the gut microbiota: a narrative synthesis of the association and recommendations
Source: Gut Microbes. 2026 Jan 31;18(1):2623356. doi: 10.1080/19490976.2026.2623356 (PMC12867399; doi:10.1080/19490976.2026.2623356)
Supplement: Supplementary_Materials_Figures.docx [file KGMI_A_2623356_SM2913.docx]

Supplementary Figures

Contents

[**Figure 1**. Overview of key gut microbiome metrics 2](#_Toc219021997)

[**Figure 2A**. Overall phylum-level taxonomic associations with SEP 3](#_Toc219021998)

[**Figure 2B.** Overall class-level taxonomic associations with SEP. 4](#_Toc219021999)

[**Figure 2C.** Overall order-level taxonomic associations with SEP. 5](#_Toc219022000)

[**Figure 2D.** Overall family-level taxonomic associations with SEP. 6](#_Toc219022001)

[**Figure 2E**. Overall genus-level taxonomic associations with SEP. 7](#_Toc219022002)

[**Figure 2F**. Overall species-level taxonomic associations with SEP. 8](#_Toc219022003)

[**Figure 3A.** Phylum-level taxonomic associations with SEP stratified by HICs and LMICs. 9](#_Toc219022004)

[**Figure 3B**. Class-level taxonomic associations with SEP stratified by HICs and LMICs 10](#_Toc219022005)

[**Figure 3C**. Order-level taxonomic associations with SEP stratified by HICs and LMICs 11](#_Toc219022006)

[**Figure 3D**. Species-level taxonomic associations with SEP stratified by HICs and LMICs 12](#_Toc219022007)

[**Figure 4A**. Phylum-level taxonomic associations with SEP stratified by life stage. 13](#_Toc219022008)

[**Figure 4B.** Class-level taxonomic associations with SEP stratified by life stage. 14](#_Toc219022009)

[**Figure 4C**. Order-level taxonomic associations with SEP stratified by life stage. 15](#_Toc219022010)

[**Figure 4D**. Family-level taxonomic associations with SEP stratified by life stage. 16](#_Toc219022011)

[**Figure 4E**. Species-level taxonomic associations with SEP stratified by life stage. 17](#_Toc219022012)

[**Figure 5**. SEP-alpha diversity associations by life stage 18](#_Toc219022013)

[**Figure 6.** SEP-beta diversity associations by life stage 19](#_Toc219022014)

[**S1.** Full systematic search. 20](#_Toc219022015)


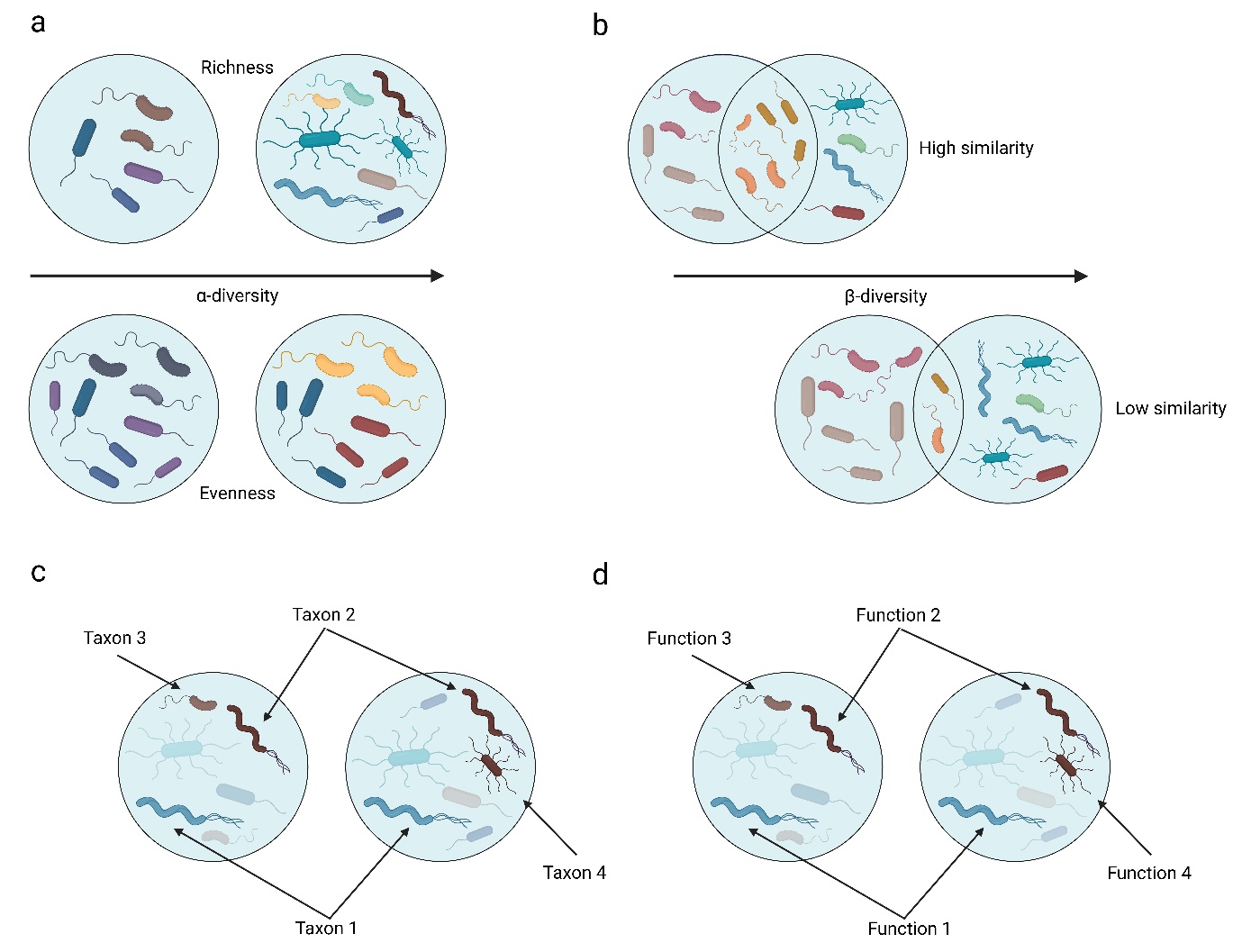


**Figure 1**. Overview of key gut microbiome metrics: (a) α-diversity quantifies diversity within a single sample; (b) β-diversity quantifies variation in composition between individuals or groups, with β-diversity increasing with dissimilarity; (c) taxonomic composition – presence and abundance of specific microbial taxa; (d) functional composition – presence and abundance of microbial pathways. Created with BioRender.


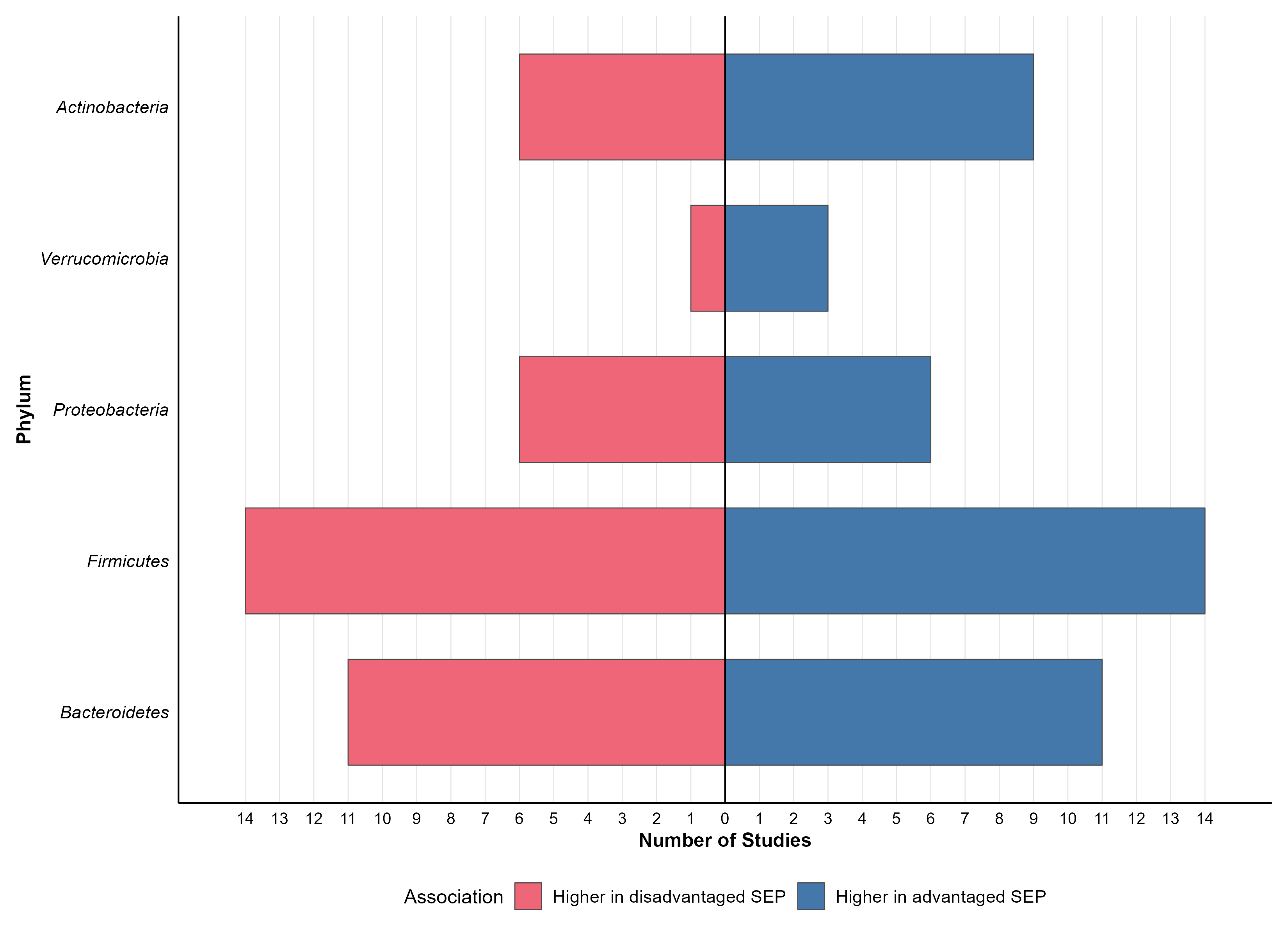


**Figure 2A**. Overall phylum-level taxonomic associations with SEP. Blue: higher abundance in advantaged SEP; red: higher in disadvantaged SEP. Only taxa reported in ≥2 studies shown.


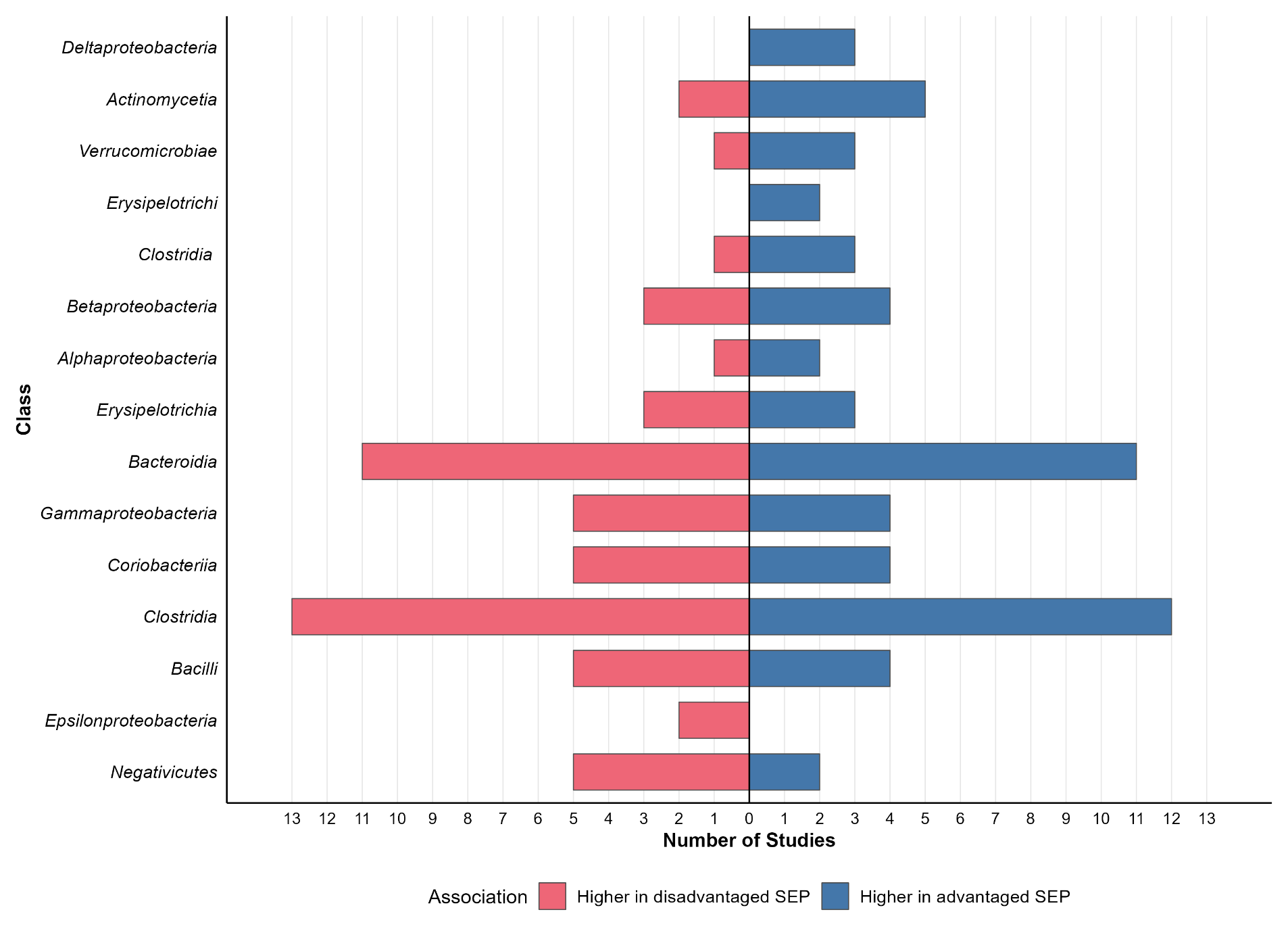


**Figure 2B.** Overall class-level taxonomic associations with SEP. Blue: higher abundance in advantaged SEP; red: higher in disadvantaged SEP. Only taxa reported in ≥2 studies shown.


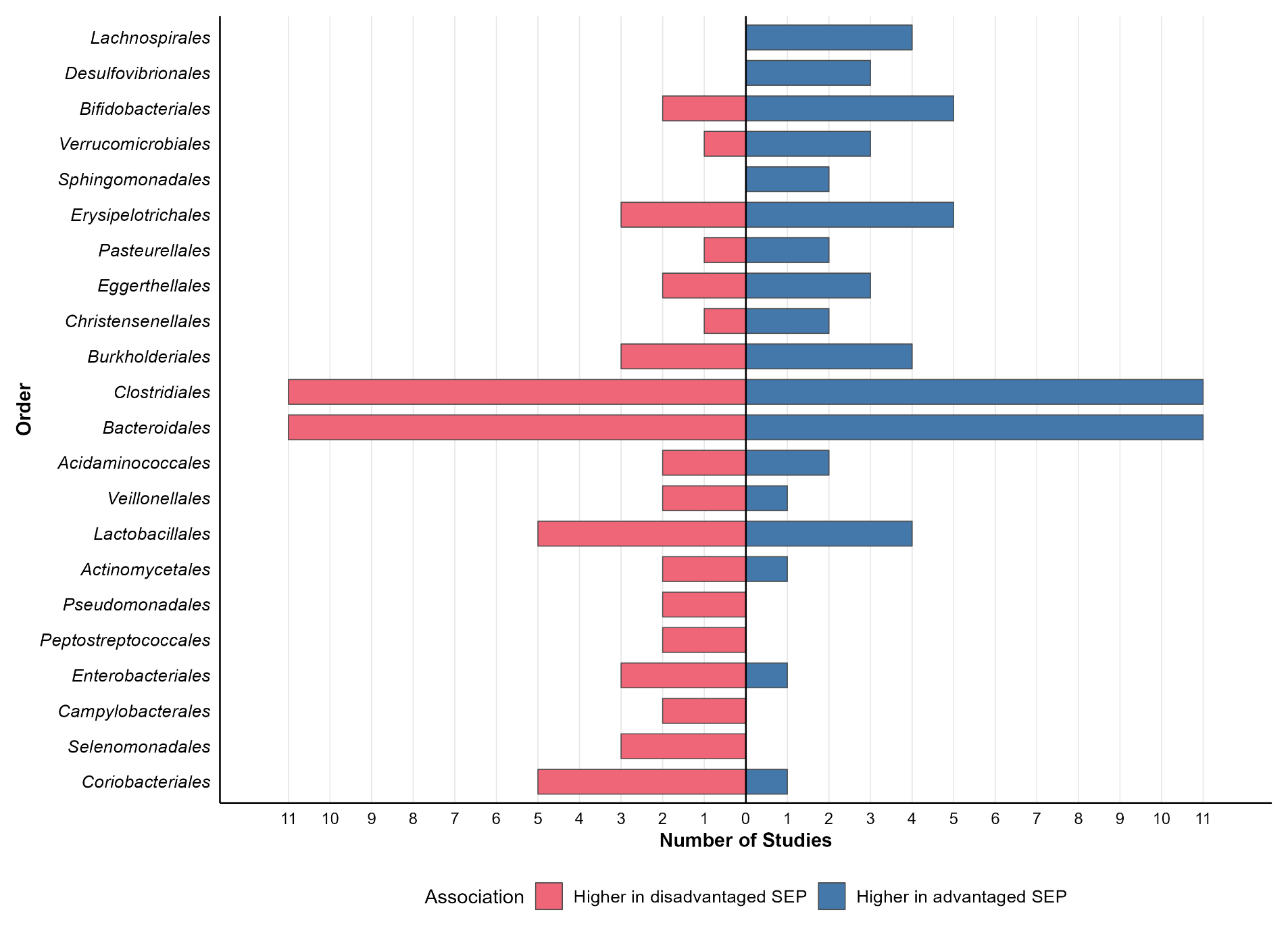


**Figure 2C.** Overall order-level taxonomic associations with SEP. Blue: higher abundance in advantaged SEP; red: higher in disadvantaged SEP. Only taxa reported in ≥2 studies shown.


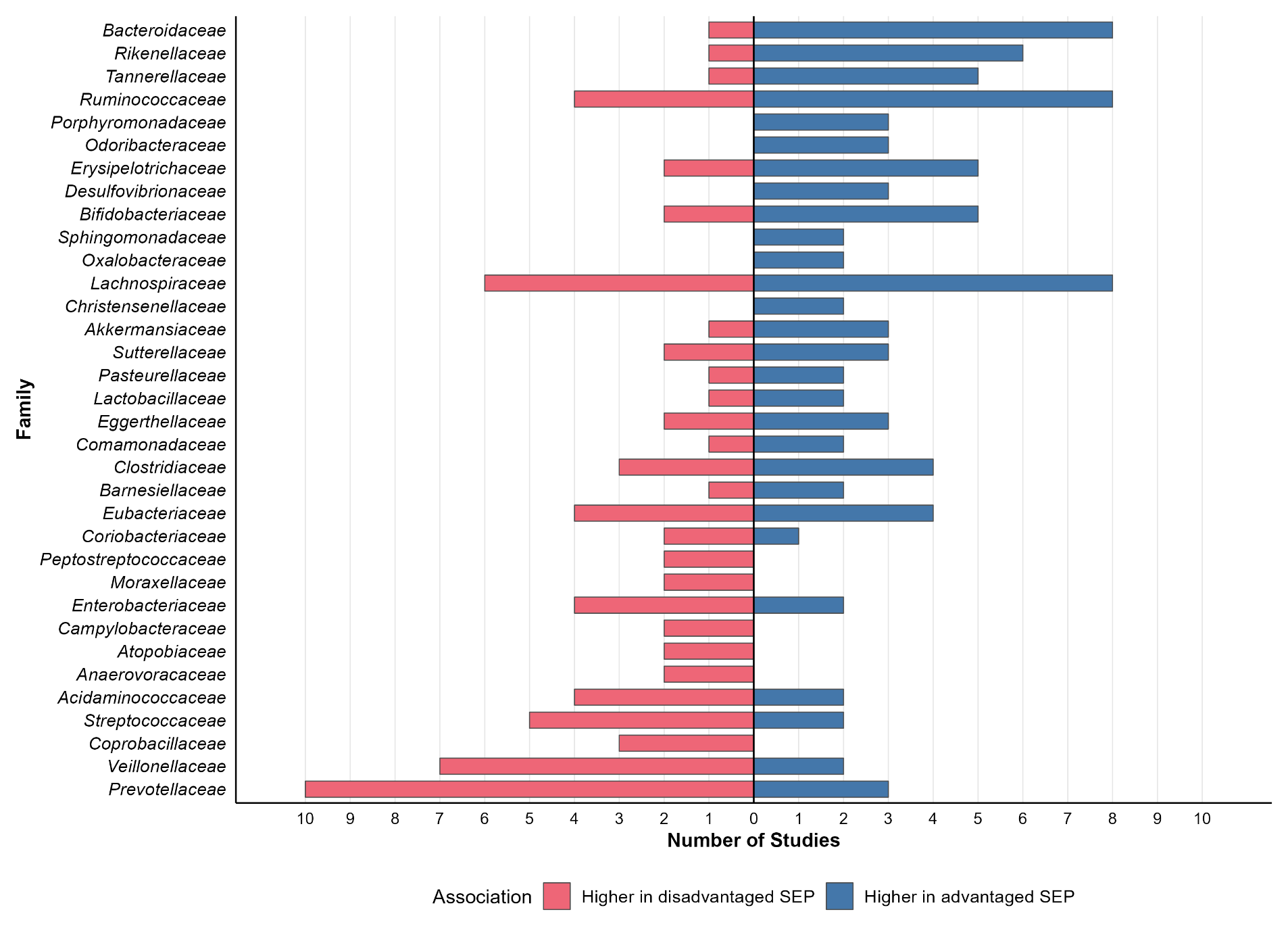


**Figure 2D.** Overall family-level taxonomic associations with SEP. Blue: higher abundance in advantaged SEP; red: higher in disadvantaged SEP. Only taxa reported in ≥2 studies shown.


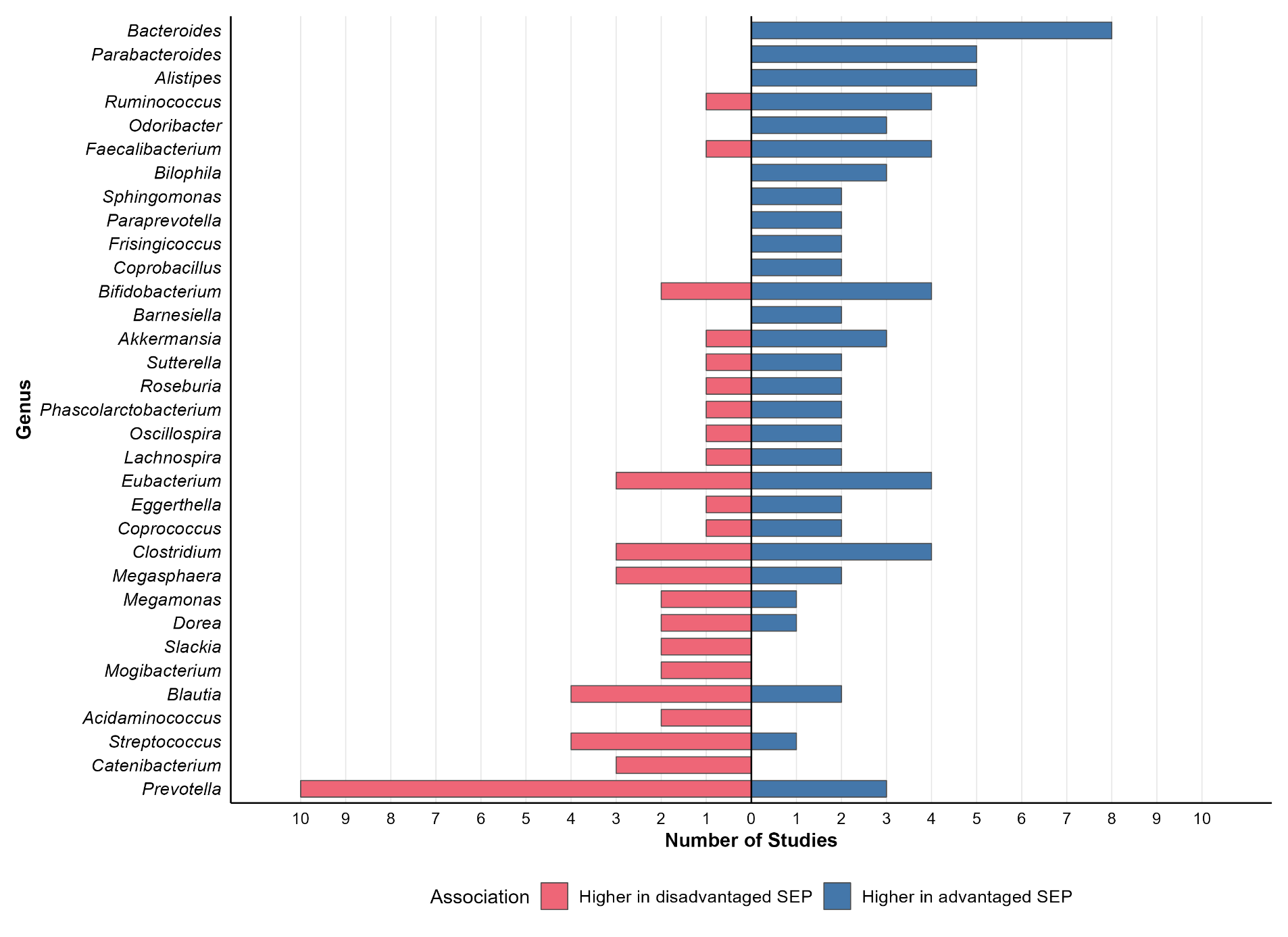


**Figure 2E**. Overall genus-level taxonomic associations with SEP. Blue: higher abundance in advantaged SEP; red: higher in disadvantaged SEP. Only taxa reported in ≥2 studies shown.


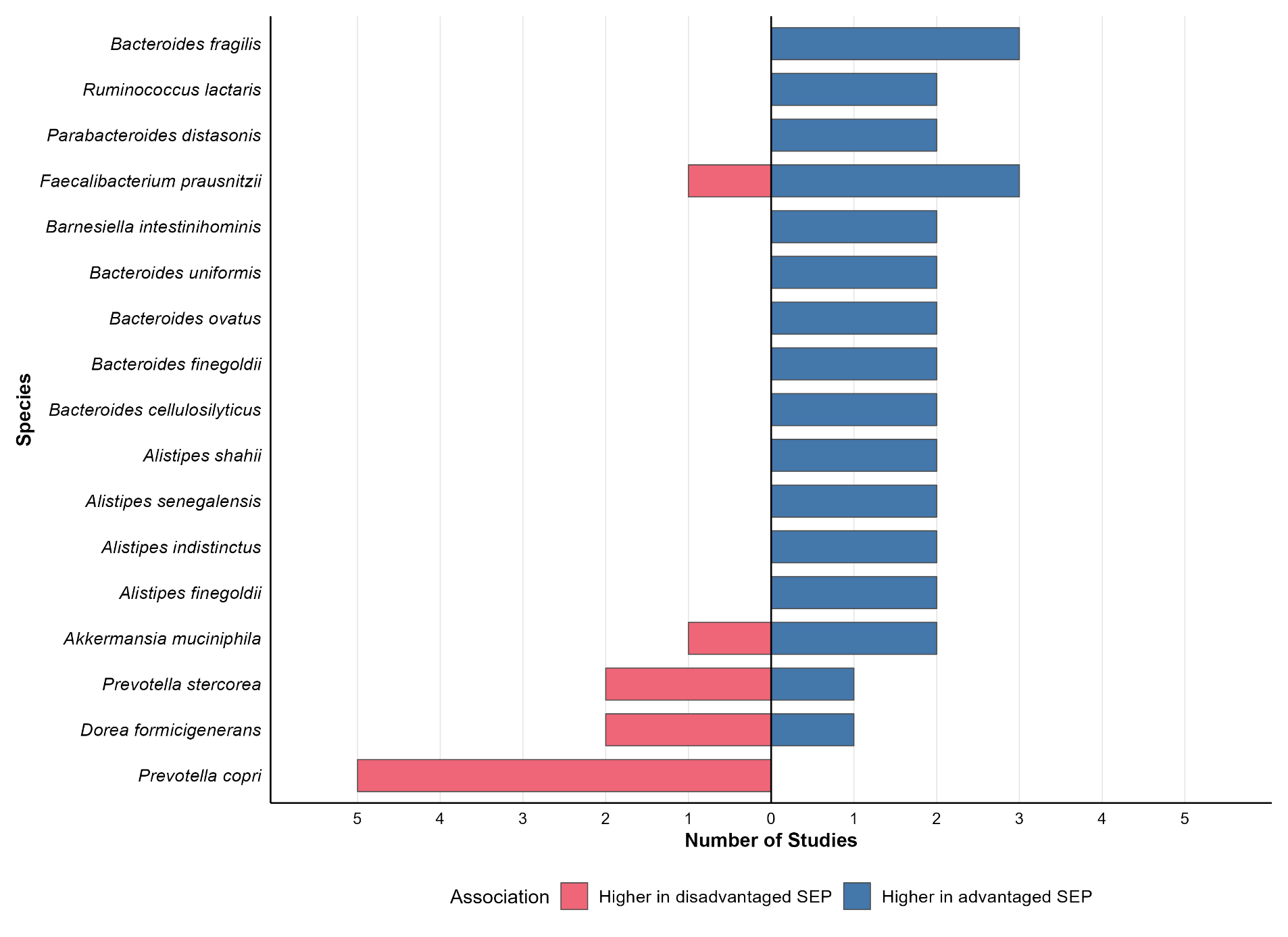


**Figure 2F**. Overall species-level taxonomic associations with SEP. Blue: higher abundance in advantaged SEP; red: higher in disadvantaged SEP. Only taxa reported in ≥2 studies shown.


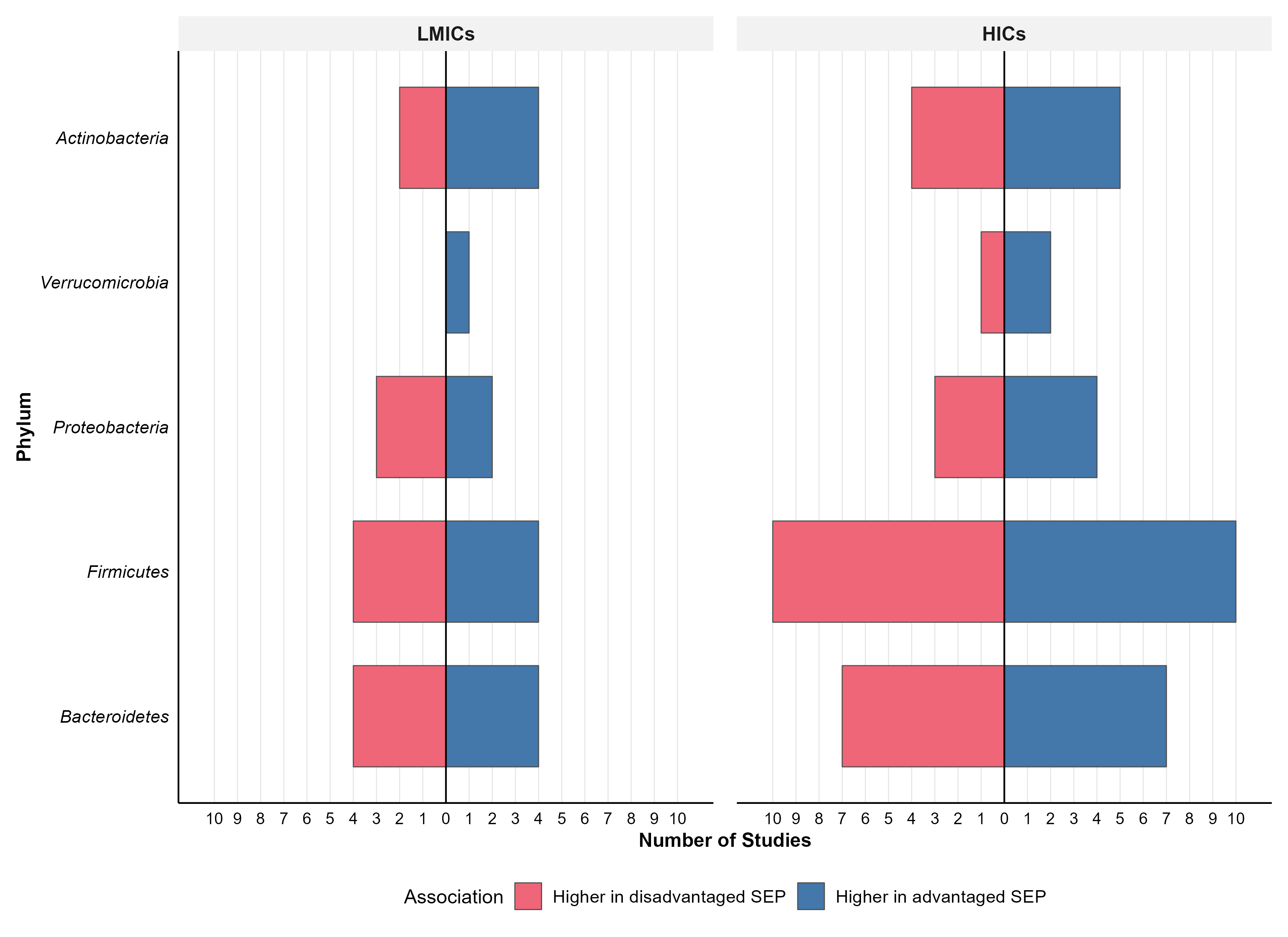


**Figure 3A.** Phylum-level taxonomic associations with SEP stratified by HICs and LMICs. Blue: higher abundance in advantaged SEP; red: higher in disadvantaged SEP. Only taxa reported in ≥2 studies shown.


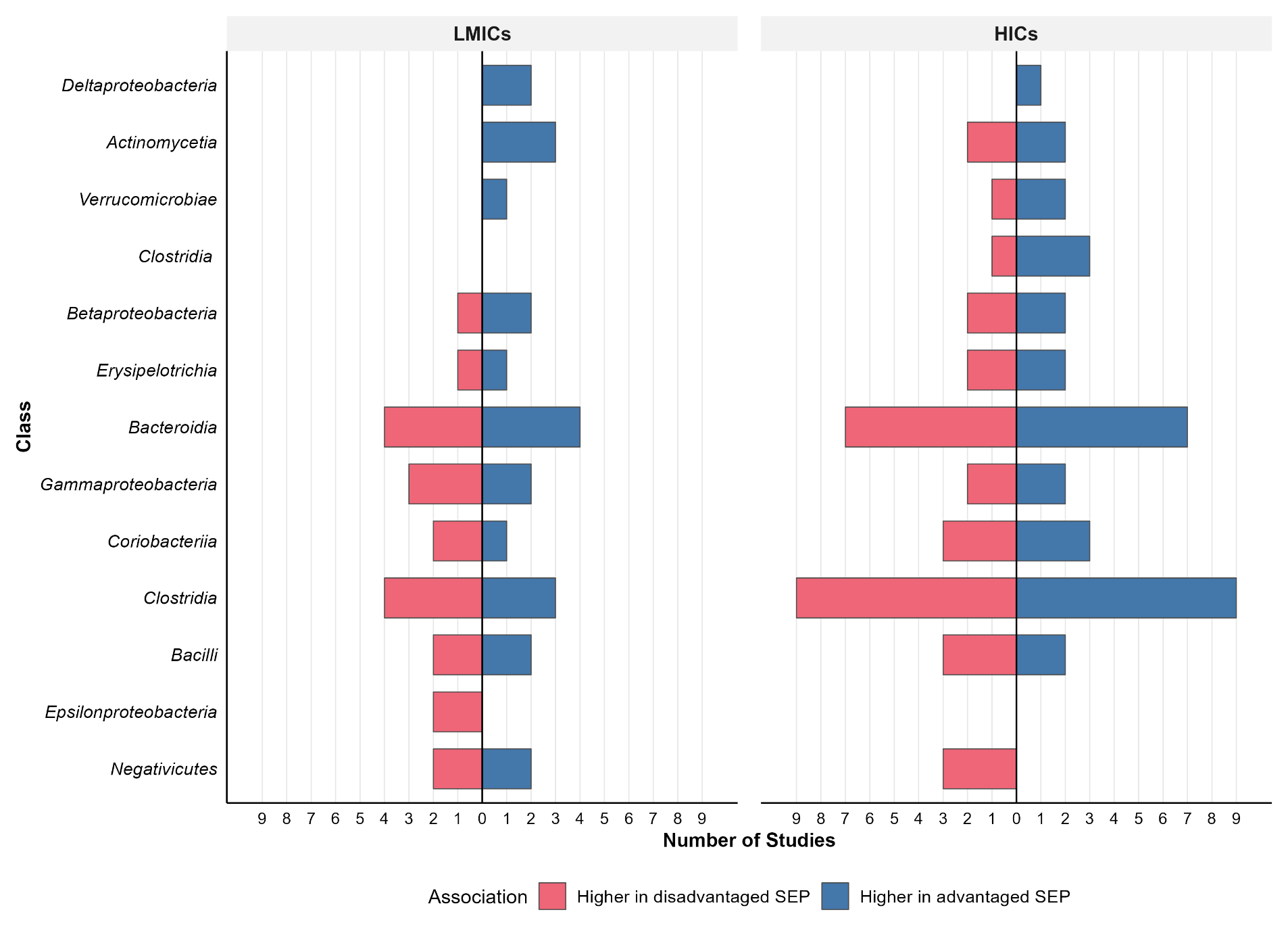


**Figure 3B**. Class-level taxonomic associations with SEP stratified by HICs and LMICs. Blue: higher abundance in advantaged SEP; red: higher in disadvantaged SEP. Only taxa reported in ≥2 studies shown.


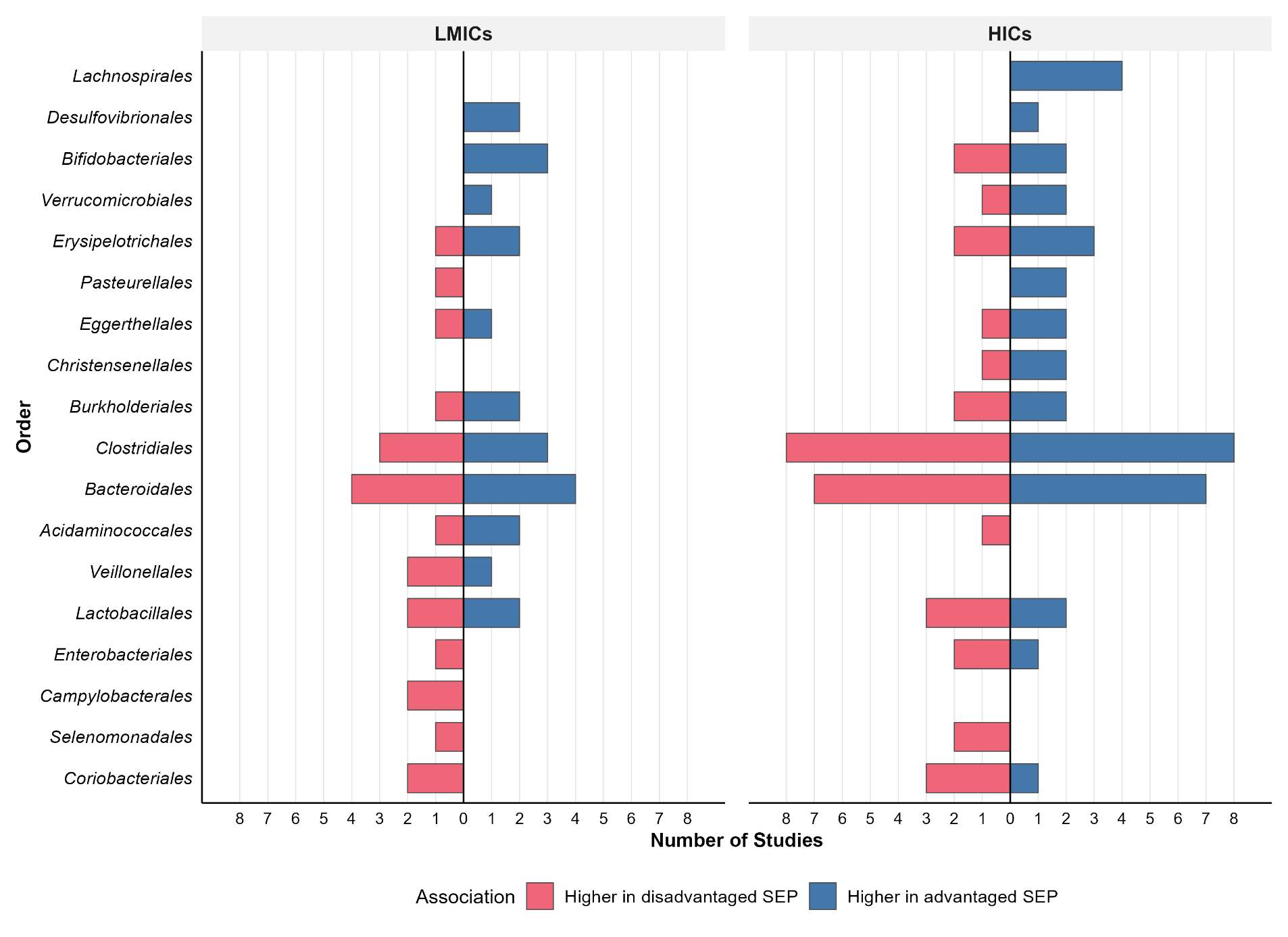


**Figure 3C**. Order-level taxonomic associations with SEP stratified by HICs and LMICs. Blue: higher abundance in advantaged SEP; red: higher in disadvantaged SEP. Only taxa reported in ≥2 studies shown.


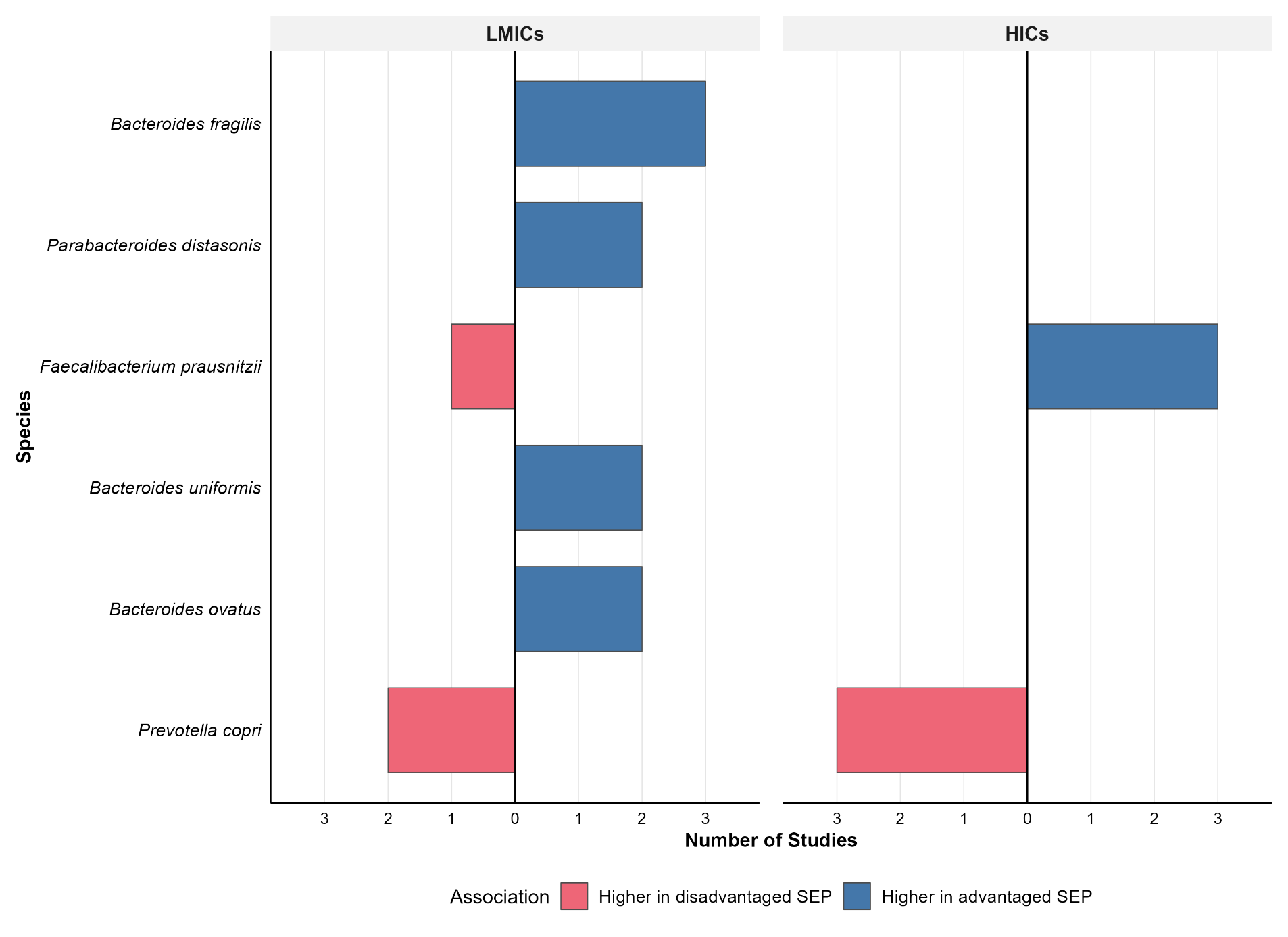


**Figure 3D**. Species-level taxonomic associations with SEP stratified by HICs and LMICs. Blue: higher abundance in advantaged SEP; red: higher in disadvantaged SEP. Only taxa reported in ≥2 studies shown.


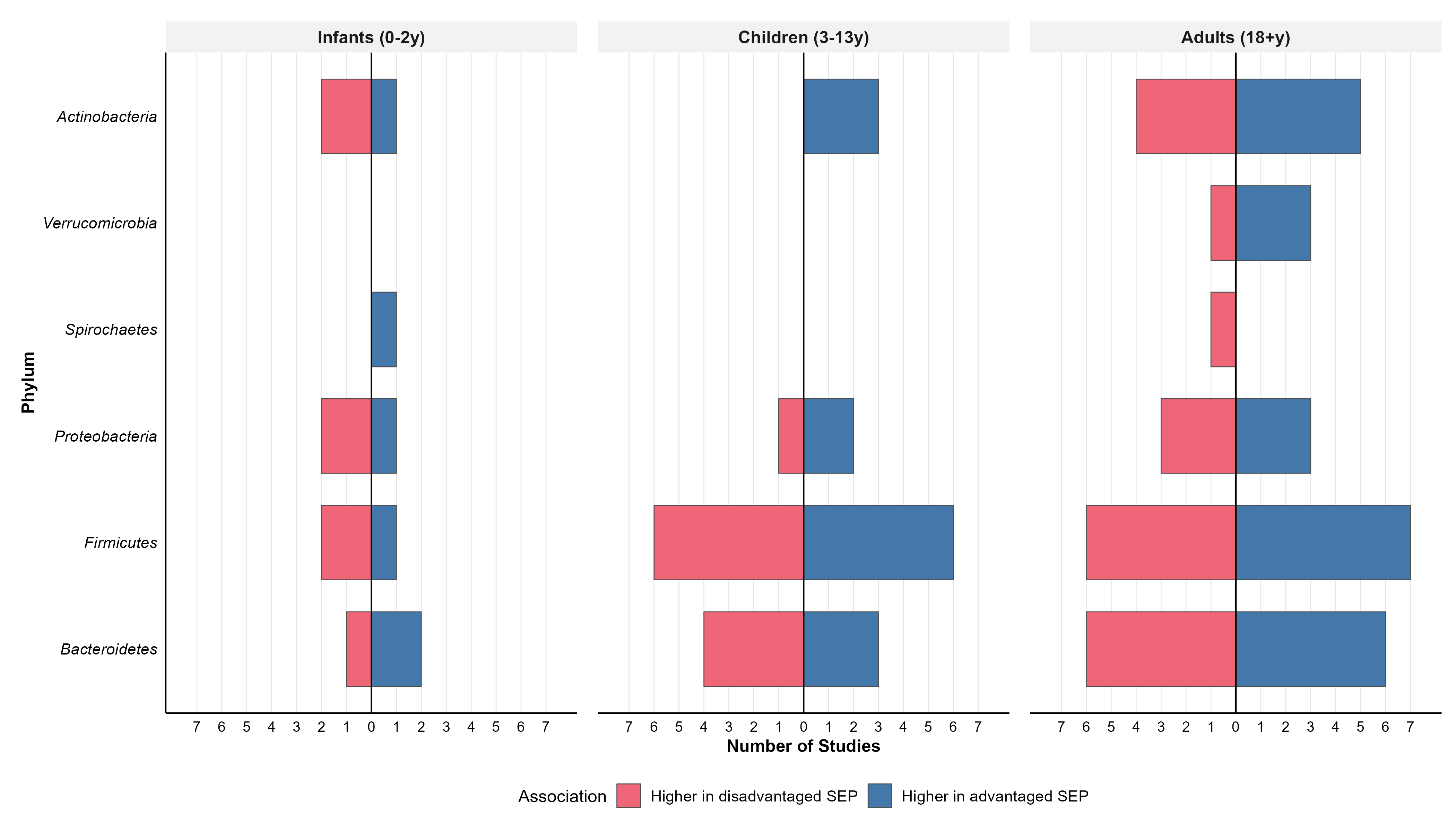
**Figure 4A**. Phylum-level taxonomic associations with SEP stratified by life stage. Blue: higher abundance in advantaged SEP; red: higher in disadvantaged SEP.


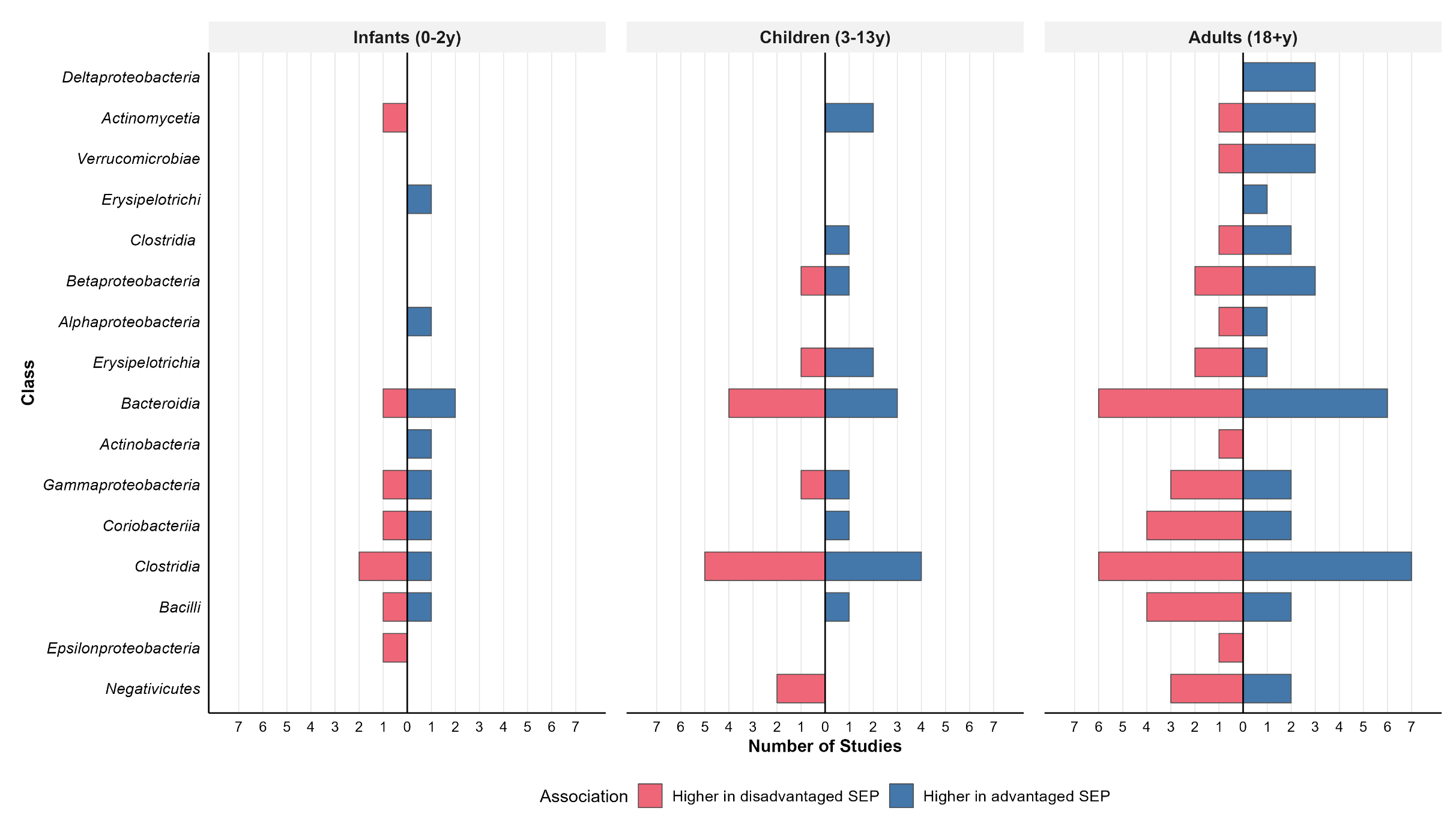
**Figure 4B.** Class-level taxonomic associations with SEP stratified by life stage. Blue: higher abundance in advantaged SEP; red: higher in disadvantaged SEP.


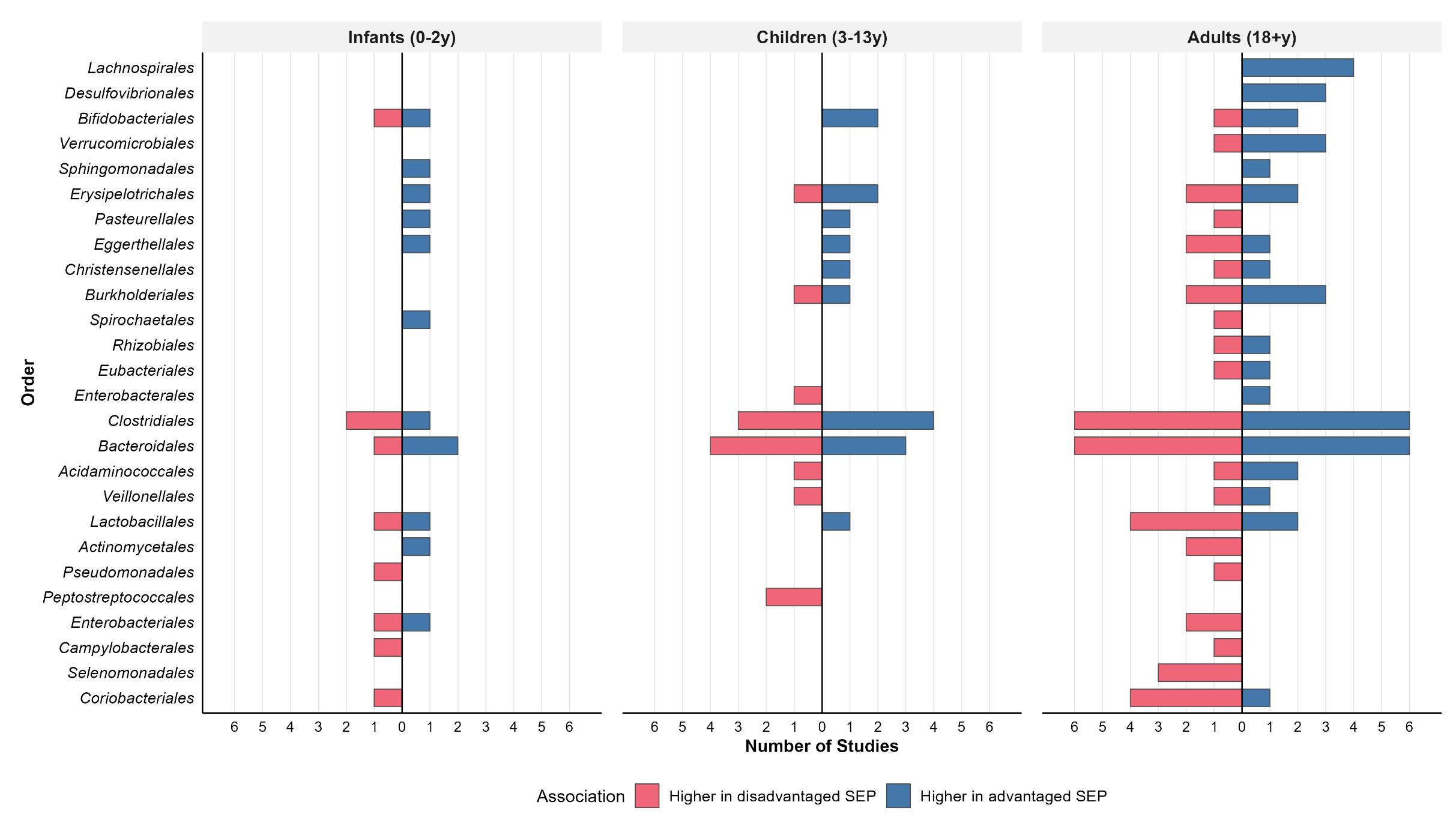
**Figure 4C**. Order-level taxonomic associations with SEP stratified by life stage. Blue: higher abundance in advantaged SEP; red: higher in disadvantaged SEP.


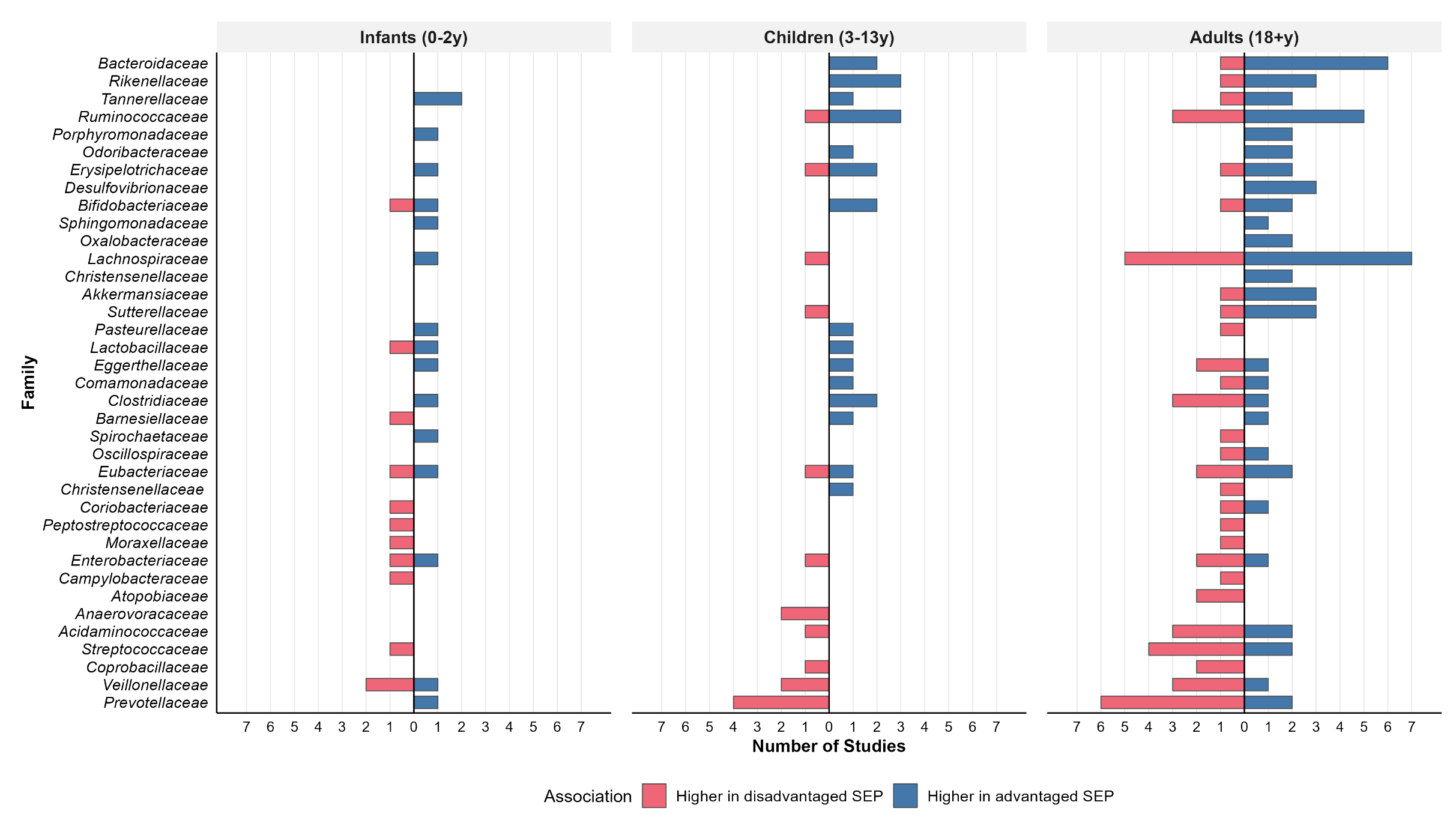
**Figure 4D**. Family-level taxonomic associations with SEP stratified by life stage. Blue: higher abundance in advantaged SEP; red: higher in disadvantaged SEP.


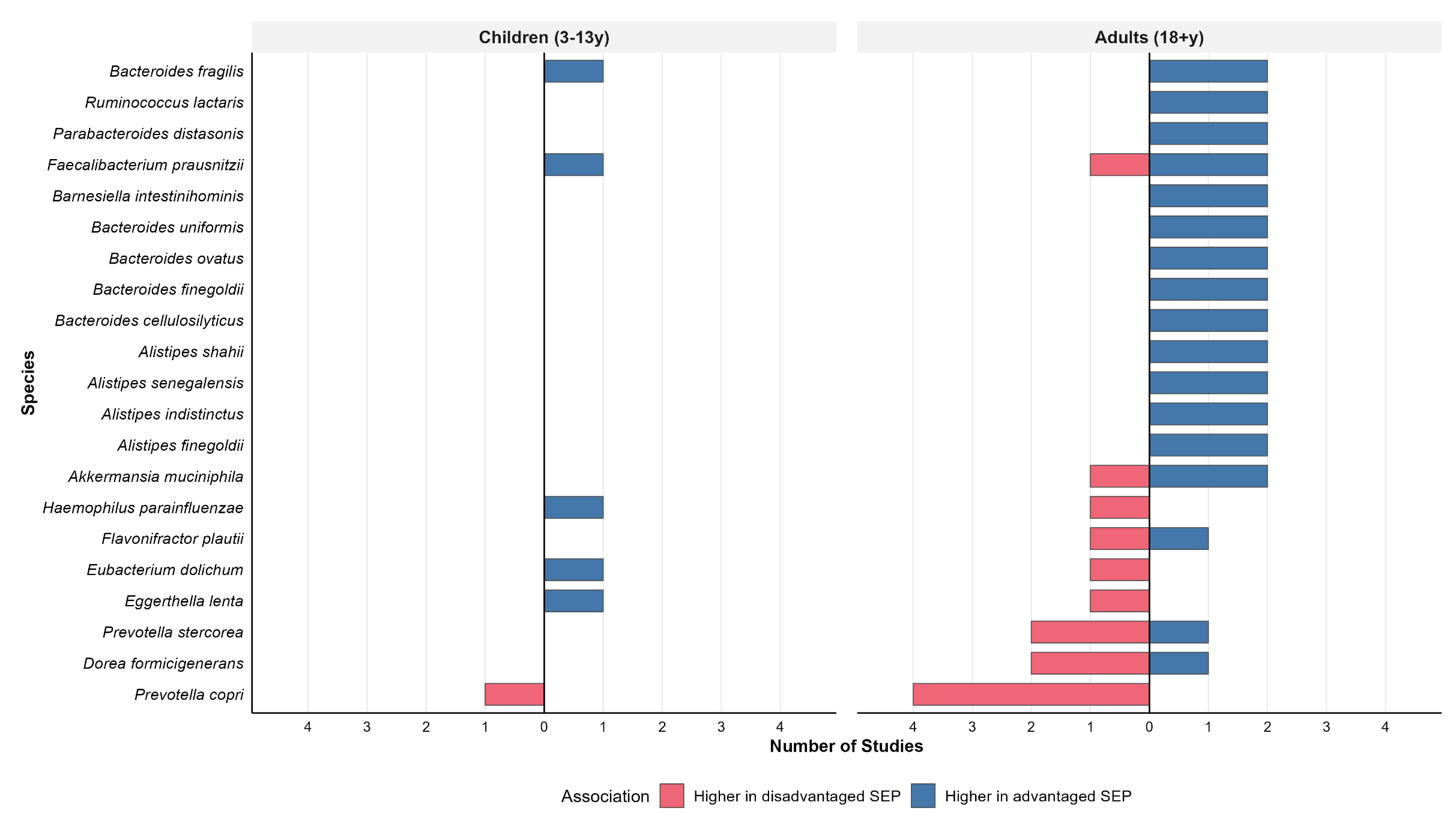
**Figure 4E**. Species-level taxonomic associations with SEP stratified by life stage. Blue: higher abundance in advantaged SEP; red: higher in disadvantaged SEP. Infancy is not shown due to no species-level associations being identified in infant studies.


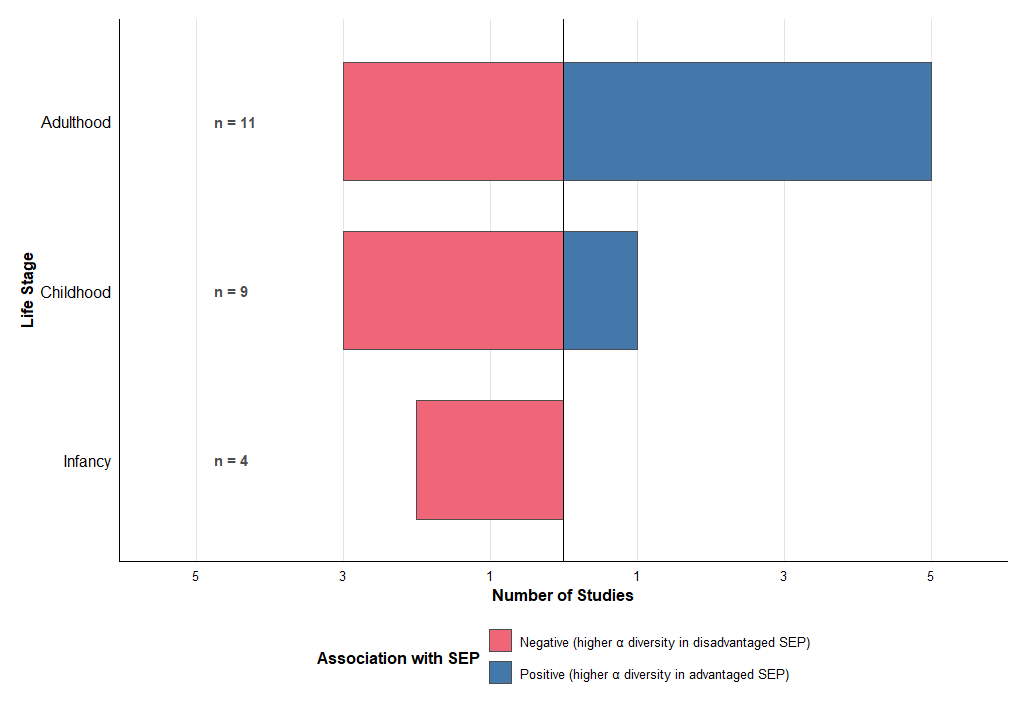


**Figure 5**. SEP-alpha diversity associations by life stage. Blue: higher alpha diversity in advantaged SEP; red: higher alpha diversity in disadvantaged SEP. Numbers on the left (n = X) represent the total studies examining alpha diversity in each life stage. Studies reporting associations dependent on other factors (e.g., obesity) or without clear directionality were excluded (n = 2).


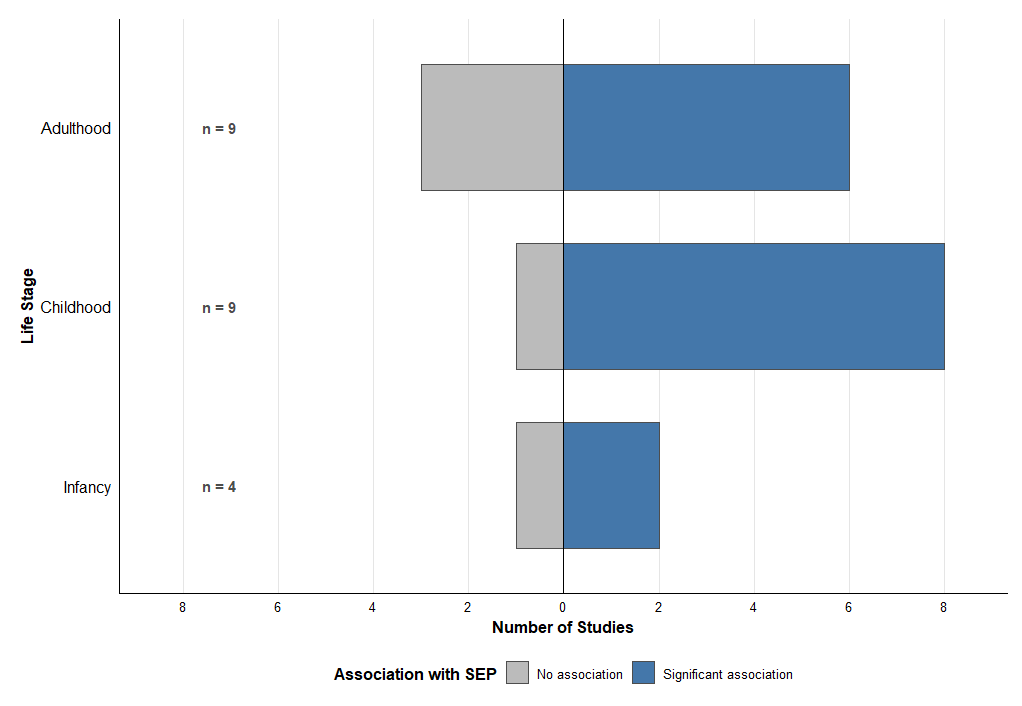


**Figure 6.** SEP-beta diversity associations by life stage. Blue: significant association; red: no association found between SEP and beta diversity. Numbers on the left (n = X) represent the total studies examining beta diversity in each life stage. Studies reporting associations dependent on other factors were excluded (n = 1).

## **S1.** Full systematic search.

("gut microbiome" OR "gut microbiota" OR "gastrointestinal microbiome" OR "Gastrointestinal Microbiome" [MeSH])

AND

("socioeconomic" OR "socioeconomic disparity" OR "socioeconomic status" OR "socioeconomic position" OR "income" OR "wealth" OR “occupation” OR “education” OR "Socioeconomic Factors" [MeSH] OR “Socioeconomic Disparities in Health” [MeSH])

AND

("sequencing" OR "16S rRNA" OR "shotgun metagenomics" OR "amplicon sequencing" OR "microbiome sequencing")
